# Integrated Omics analysis of pig muscle metabolism under the effects of dietary *Chlorella vulgaris* and exogenous enzymes

**DOI:** 10.1038/s41598-022-21466-z

**Published:** 2022-10-10

**Authors:** Diogo Coelho, David Ribeiro, Hugo Osório, André Martinho de Almeida, José António Mestre Prates

**Affiliations:** 1 CIISA - Centro de Investigação Interdisciplinar Em Sanidade Animal, Faculdade de Medicina Veterinária, Universidade de Lisboa, Alto da Ajuda, 1300-477 Lisbon, Portugal; 2 Laboratório Associado Para Ciência Animal E Veterinária (AL4AnimalS), Lisbon, Portugal; 3 LEAF - Linking Landscape, Environment, Agriculture and Food Research Center, Associated Laboratory TERRA, Instituto Superior de Agronomia, Universidade de Lisboa, Tapada da Ajuda, 1349-017 Lisbon, Portugal; 4 i3S - Instituto de Investigação E Inovação Em Saúde, Universidade Do Porto, 4200-135 Porto, Portugal; 5 IPATIMUP - Institute of Molecular Pathology and Immunology of the University of Porto, Universidade Do Porto, 4200-135 Porto, Portugal; 6 Departamento de Patologia, Faculdade de Medicina, Universidade Do Porto, 4200-319 Porto, Portugal

**Keywords:** Biochemistry, Computational biology and bioinformatics, Molecular biology, Zoology

## Abstract

Monogastric feeding is dependent on costly conventional feedstuffs. Microalgae such as *Chlorella vulgaris* are a sustainable alternative; however, its recalcitrant cell wall hinders monogastric digestion. Carbohydrate Active Enzyme (CAZyme) supplementation is a possible solution. The objective of this work was to evaluate the effect of 5% dietary *C. vulgaris* (CV) and enzymatic supplementation (CV + R—Rovabio® Excel AP; CV + M—four CAZyme mix) on muscle transcriptome and proteome of finishing pigs, in an integrated approach. Control pigs increased the abundance of contractile apparatus (MYH1, MYH2, MYH4) and energy metabolism (CKMT1, NDUFS3) proteins, demonstrating increased nutrient availability. They had increased expression of *SCD*, characteristic of increased glucose availability, via the activation of SREBP-1c and ChREBP. CV and CV + R pigs upregulated proteolytic and apoptotic genes (*BAX*, *DDA1*), whilst increasing the abundance of glucose (UQCRFS1) and fatty acid catabolism (ACADS) proteins. CV + R pigs upregulated *ACOT8* and *SIRT3* genes as a response to reduced nutrient availability, maintaining energy homeostasis. The cell wall specific CAZyme mix, CV + M, was able to comparatively reduce Omics alterations in the muscle, thereby reducing endogenous nutrient catabolism compared to the CV + R and CV.

## Introduction

The resources and sustainability of our planet will come under high pressure due to the increase of the worldwide human population, expected to surpass 9 billion by 2050^1^. This, together with the income increase of developing countries will trigger an increased demand for meat products, including pork^2^. Pig production is largely dependent on intensive production systems that use corn and soybean meal as major dietary sources of energy and crude protein, respectively^1,3^. However, the production and transport of these feedstuffs have negative environmental and economic impacts as it requires large arable land areas, water and pesticides and are costly to transport^2,3^.

Pork presents an unhealthy perception due to the lower proportions of polyunsaturated fatty acids (PUFA) and lipid-soluble antioxidant vitamins, with higher percentages of saturated fatty acids (SFA)^4,5^. In fact, a large part of the human population does not consume the recommended levels of *n*-3 PUFA by World Health Organization (WHO), particularly eicosapentaenoic acid (EPA, 20:5*n*-3) and docosahexaenoic acid (DHA, 22:6*n*-3) the two most important health-promoting *n*-3 PUFA^6,7^. Nevertheless, it is well established that diet provides an effective approach for altering pork’s fat composition and nutritional value^8^.

It is therefore imperative to find sustainable alternatives to conventional feedstuffs with an interesting content in *n*-3 PUFA^7,9^. One of such alternatives are microalgae, a valuable aquatic resource, due to their environment-friendly production and interesting nutritional composition^10,11^. *Chlorella vulgaris* stands out among microalga species^12^. It is a freshwater unicellular eukaryotic green microalga, that is grown worldwide, and it is known for its high biomass productivity, relatively easy cultivation and balanced nutritional composition^12,13^. These characteristics make *C. vulgaris* an interesting alternative for monogastric diets, namely pigs^13^. In particular, *C. vulgaris* has an interesting content in some *n*-6 PUFA (18:2*n*-6 and 18:3*n*-6) and in the essential α-linolenic acid (ALA, 18:3*n*-3), albeit with lower amounts of EPA and DHA^14^. Moreover, *C. vulgaris* presents a high content in different functional bioactive compounds, including antioxidant carotenoids^12^.

Conversely, it has a recalcitrant cell wall composed by a diverse and complex matrix of cross-linked insoluble carbohydrates^15^. Consequently, the bioavailability of its valuable nutrients and their absorption by pigs is compromised in high dietary incorporation levels of *C. vulgaris*^16,17^. The use of exogenous feed enzymes is a proposed strategy to overcome the problem of the cell wall recalcitrant nature^18^, including the introduction of carbohydrate-active enzymes (CAZymes) in monogastric diets to improve feed nutritive value and enhance animal performance and health^19^. Several in vitro studies have demonstrated the ability of CAZymes to degrade microalgae cell walls^20–22^. In 2019, Coelho and colleagues^23^ developed a *C. vulgaris* cell wall disruption in vitro assay and identified an enzymatic mixture (composed by an *exo*-β-glucosaminidase, an alginate lyase, a peptidoglycan N-acetylmuramic acid deacetylase and a lysozyme) with potential to disrupt the cell wall in vivo and releasing inaccessible nutrients with important nutritional value.

The use of *C. vulgaris* in pig diets is mostly reported at low incorporation levels, namely as a feed supplement (< 1% in the diet)^2^. In addition, such published studies do not assess how a high dietary incorporation level of *C. vulgaris* affects the pig metabolism, mainly lipid and oxidative stress molecular pathways, since *C. vulgaris* is rich in ALA, the precursor of EPA and DHA on *n*-3 long chain (LC) PUFA biosynthesis pathway^24^ and antioxidant carotenoids^12^, respectively.

Omics, such as transcriptomics and proteomics, enable studying the metabolism of pigs under the influence of external factors^25^, leading to the emergence of novel disciplines, such as foodomics^26^ and nutrigenomics^27^. Thus, it allows studying changes caused by diet in meat quality, at a molecular level^28,29^. For instance, conversion of muscle into meat has been thoroughly described using proteomics^30^, whereas transcriptomics has been used to study the effect of dietary linseed, with or without vitamin E, in the pig muscle^31^. Combining and integrating both approaches allow studying gene-protein interactions, which has been performed to compare skeletal muscle metabolism of Chinese and western pig breeds^32^ and to investigate the metabolism of *longissimus dorsi* of double-muscled Large White pigs^33^.

The aim of this study was to evaluate the influence of a high dietary incorporation level of *C. vulgaris*, and of two exogenous CAZyme mixtures (the commercially available Rovabio® Excel AP (Adisseo, Antony, France) and the four-CAZyme mixture developed by Coelho et al*.*^23^), on muscle transcriptome and proteome profiles, focusing on lipid and oxidative stress metabolism, of finishing pigs.

## Results

### Animal performance and *longissimus lumborum* meat quality and composition

These results have been reported in a companion paper^34^ and are mentioned here for contextualization purposes. Growth performance, carcass characteristics and meat quality traits were not significantly affected by dietary treatments. Pigs fed with *C. vulgaris* diets significantly increased total carotenoid deposition. For instance, CV + R increased the total chlorophylls and carotenoids by 2.1-fold compared to control. Microalga diets significantly increased *n*-3 PUFA and reduced the *n*-6:*n*-3 ratio in meat, thanks to increases in *n*-3 PUFA including 18:3*n*-3, 20:3*n*-3, 20:5*n*-3, 22:5*n*-3, and 22:6*n*-3.

### Sequencing output and identification of expressed transcripts in the pig muscle transcriptome

In this study, we performed the analysis of the whole muscle transcriptome of each finishing pig fed with the following experimental diets: Control, CV, CV + R and CV + M. The total number of reads generated by RNA-Seq are reported in Supplementary Table S1. The average number of raw reads per experimental group was 65,140,430 for control, 38,830,084 for CV, 31,702,033 for CV + R and 30,641,589 for CV + M. Raw reads were trimmed, generating the average numbers of reads per sample for each experimental group of 45,219,804.5 (69.4%), 34,349,352.17 (88.5%), 30,768,719.67 (97.1%) and 27,093,411 (88.4%) for Control, CV, CV + R and CV + M, respectively. The trimmed reads were used for further analysis.

The trimmed reads were mapped in the *Sus scrofa* genome using hisat2 tool. The total average number of mapped reads was 40,839,191 (89.5%) for control, 30,977,391 (90%) for CV, 28,155,015 (91.5%) for CV + R and 24,558,272 (90.7%) for CV + M, while the average of uniquely mapped reads was 34,293,067 (74.7%) for control, 28,293,700 (81.3%) for CV, 25,692,735 (83.1%) for CV + R, and 21,706,256 (79.7%) for CV + M. The density of the mapped reads on different regions of the genome is displayed in Supplementary Figure S1.

The gene expression was quantified using the featureCounts tool, which reports the raw counts of reads that map to a single location in each of the exons (feature) that belong to that gene^35^. The top ten most abundantly expressed coding genes of muscle transcriptome in all experimental groups (raw counts from 340,811 to 1,219,730), ranked by absolute abundance were: ATP synthase F0 subunit 6 (*ATP6*), Cytochrome c oxidase subunit III (*COX3*), Nebulin (*NEB*), Enolase 3 (*ENO3*), NADH dehydrogenase subunit 4 (*ND4*), Myosin heavy chain 1 skeletal muscle (*MYH1*), Lactate dehydrogenase A (*LDHA*), Actin alpha 1 skeletal muscle (*ACTA1*), Myosin light chain phosphorylable fast skeletal muscle (*MYLPF*), and Myosin light chain 1 (*MYL1*) (Supplementary Fig. S2).

### Transcriptome profile and differential expression

Among the muscle transcriptome data, we identified 781, 596, 744 and 575 genes, including protein-coding genes and ncRNA genes, that were expressed in the Control, CV, CV + R and CV + M groups, respectively. We also identified 17,811 genes that were expressed in all experimental groups and 24,584 genes that were expressed, at least, in one experimental group.

**Figure 1:**
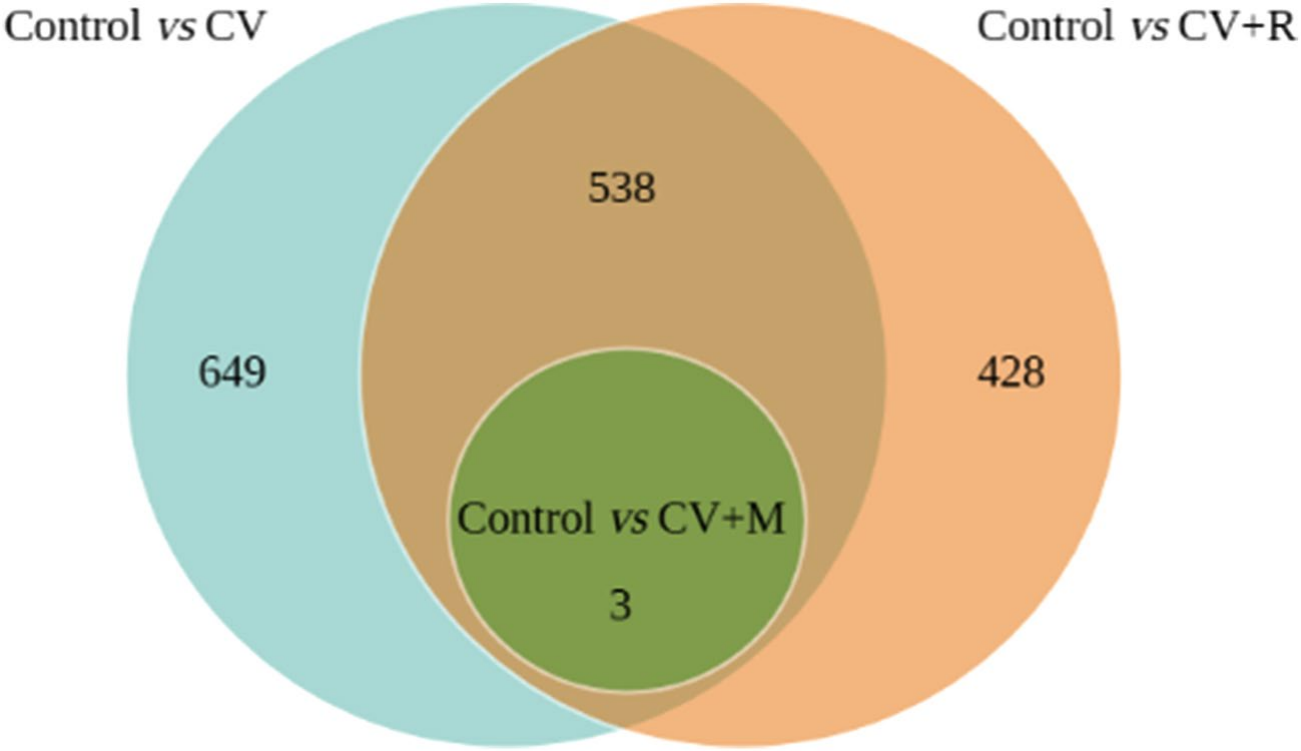
Venn diagram with the distribution of the DEGs in the three experimental diet comparisons.

The DESeq2 tool was used to test for differential gene expression analysis between the following comparisons among experimental diets: Control *vs* CV, Control *vs* CV + R, and Control *vs* CV + M. In the pool of annotated genes from pig muscle transcriptome, we identified 1190 significant DEGs (245 upregulated and 945 downregulated) in the Control *vs* CV, 969 significant DEGs (277 upregulated and 692 downregulated) in the Control *vs* CV + R, and 3 significant DEGs (2 upregulated and 1 downregulated) in the Control *vs* CV + M. The number of non-redundant DEGs is 649 for Control *vs* CV and 428 for Control *vs* CV + R, with 538 DEGs common to these two comparisons and 3 DEGs common to the three comparisons. This is represented in the Venn diagram (Fig. 1). Based on the results from the DEGs analysis, a Multi-dimensional scaling (MDS) plot was performed in order to assess how the samples are distributed in a two-dimensional analysis according to leading logFC, and also a hierarchical clustering of treatment condition samples based on Pearson and Spearman correlations. A volcano plot of DEGs, which shows the relationship between FC and evidence of differential expression (-log FDR) was also developed.

**Figure 2:**
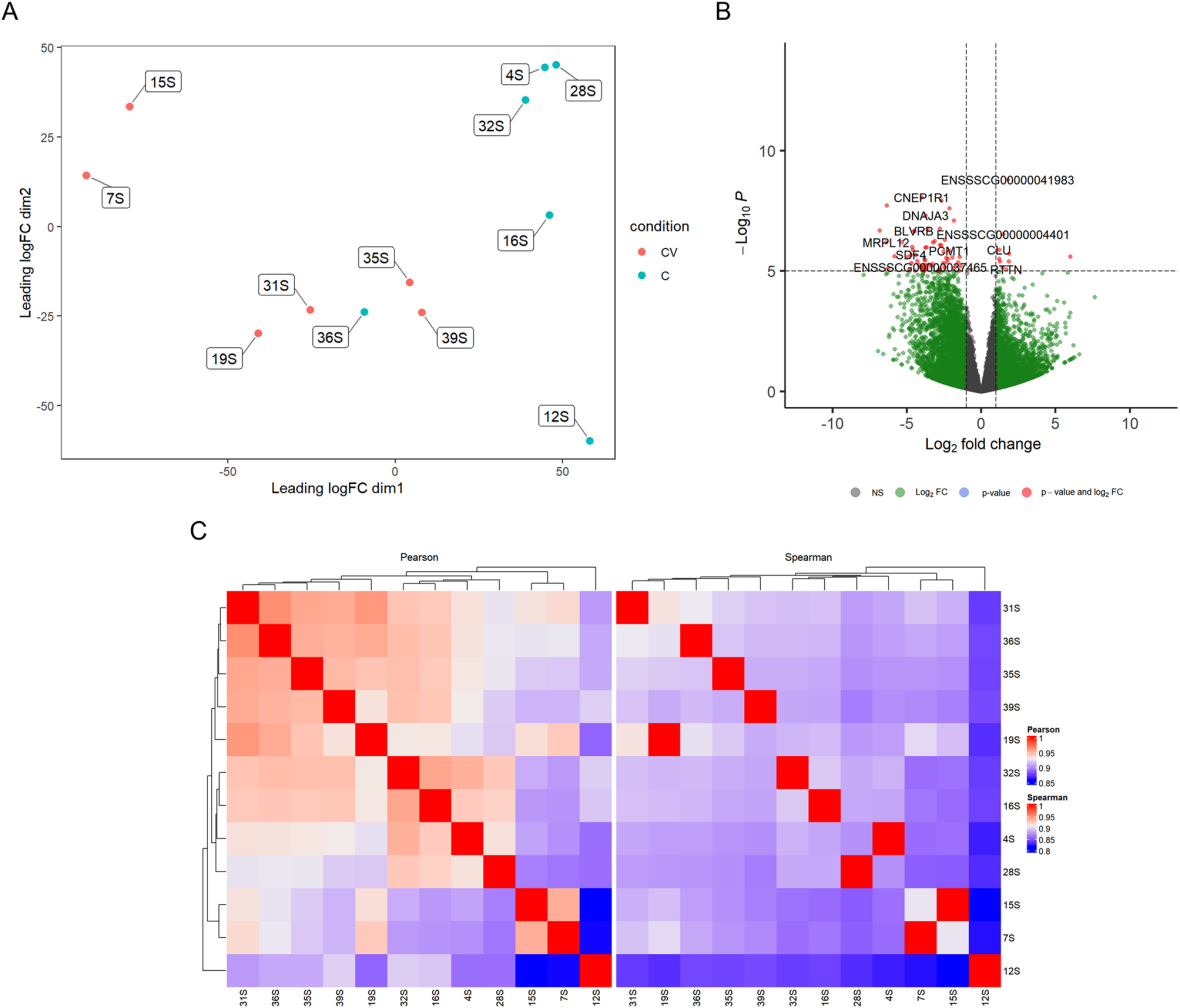
(**A**) Multi-dimensional scaling (MDS) plot showing the relation between Control and CV diet samples. (**B**) Volcano plot showing the relationship between FC and evidence of differential expression (-log FDR) for Control *vs* CV. (**C**) Clustering of sample to sample correlations—Control *vs* CV. Hierarchical clustering of treatment condition samples based on Pearson (left) and Spearman correlations (right).

In the comparison Control *vs* CV, we observed in the MDS plot a good discrimination between the two experimental groups with samples from Control to occupy higher values of leading logFC dimension 1 with the exception of the sample 36S (Fig. 2A), which is according with the correlations (Fig. 2C). The red dots in the volcano plot highlight some of the identified DEGs (Fig. 2B).

**Figure 3:**
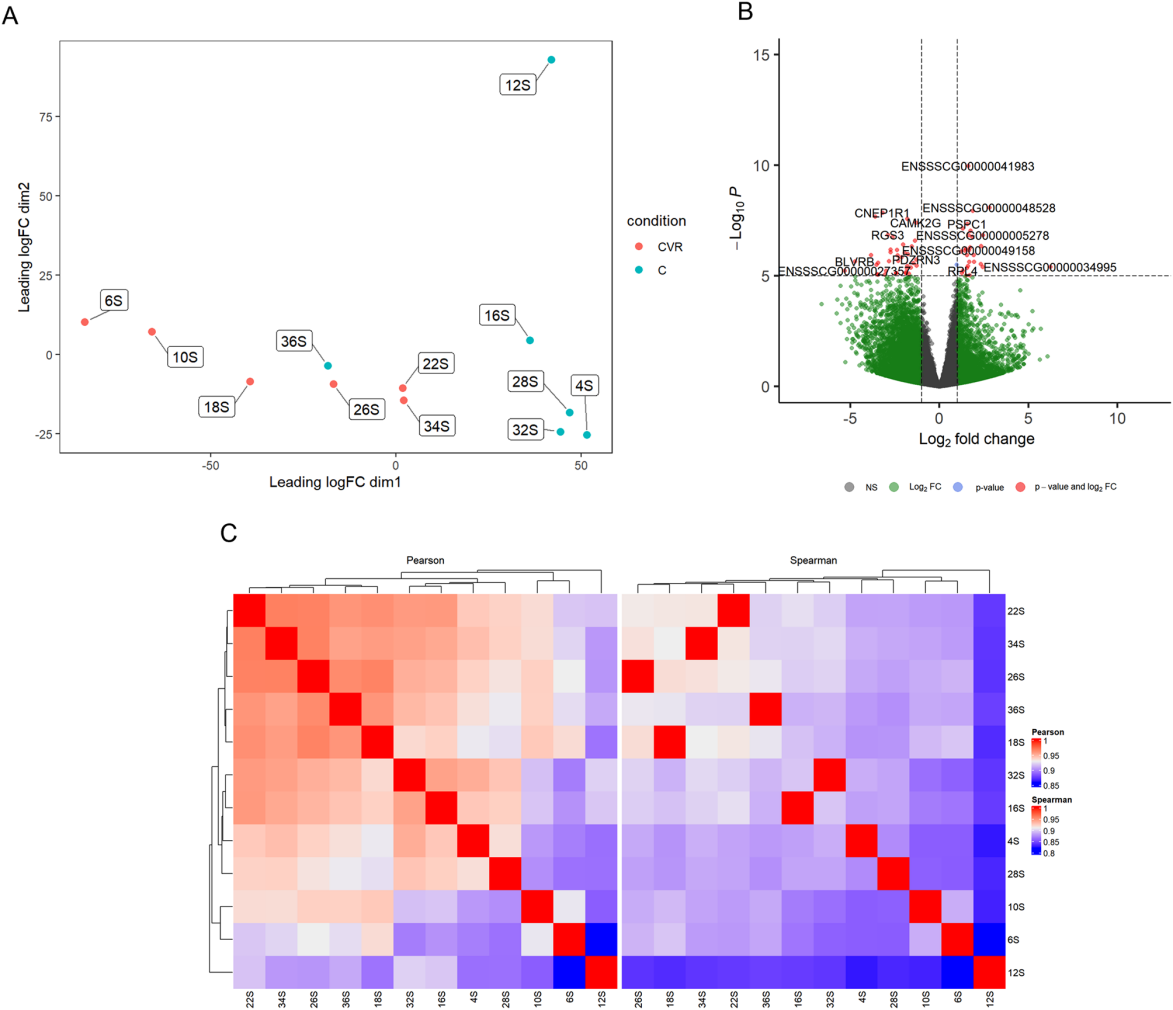
(**A**) Multi-dimensional scaling (MDS) plot showing the relation between Control and CV + R diet samples. (**B**) Volcano plot showing the relationship between FC and evidence of differential expression (-log FDR) for Control *vs* CV + R. (**C**) Clustering of sample to sample correlations—Control *vs* CV + R. Hierarchical clustering of treatment condition samples based on Pearson (left) and Spearman correlations (right).

In the comparison Control *vs* CV + R, the MDS plot also demonstrates a good discrimination between the two experimental groups, with Control located in the region corresponding to higher values of leading logFC dimension 1 and CV + R with lower values of leading logFC dimension 1 and dimension 2 (Fig. 3A). This information is corroborated by the correlation coefficients presented in Fig. 3C. Some of the identified DEGs are presented as red dots in the volcano plot (Fig. 3B).

**Figure 4:**
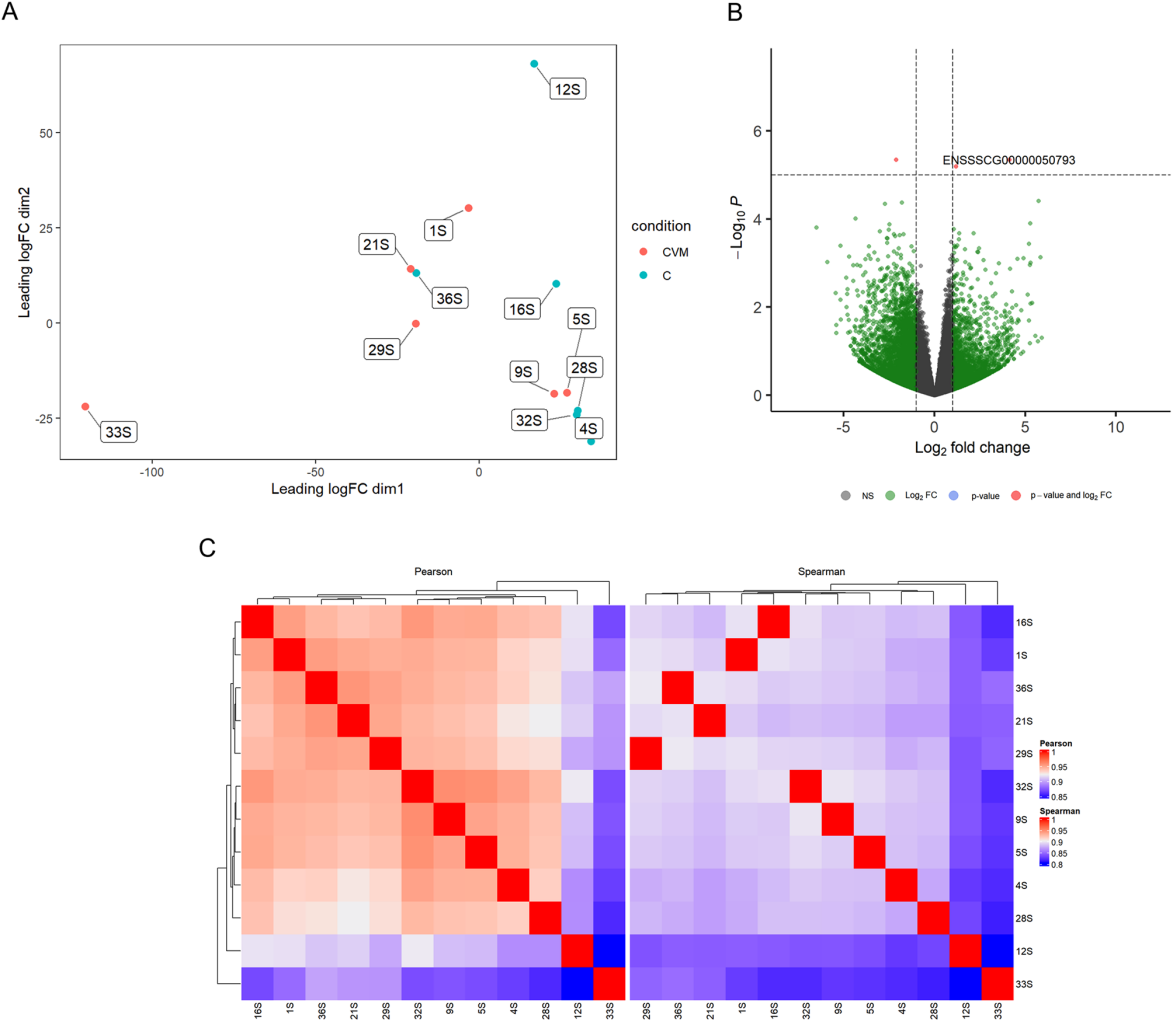
(**A**) Multi-dimensional scaling (MDS) plot showing the relation between Control and CV + M diet samples. (**B**) Volcano plot showing the relationship between FC and evidence of differential expression (-log FDR) for Control *vs* CV + M. (**C**) Clustering of sample to sample correlations—Control *vs* CV + M. Hierarchical clustering of treatment condition samples based on Pearson (left) and Spearman correlations (right).

In the comparison Control *vs* CV + M, less discrimination was observed between these two experimental groups in the MDS plot (Fig. 4A) and in the correlations (Fig. 4C), when comparing with previous comparisons. Thus, a much lower number of DEGs was identified between these two experimental groups, marked as red dots in the volcano plot (Fig. 4B) compared with Control *vs* CV and Control *vs* CV + R.

### Functional classification of DEGs

In order to understand which biological pathways and processes were represented by the identified DEGs, a functional analysis was performed. For this analysis, in comparisons Control *vs* CV and Control *vs* CV + R, were selected the 50 most up and 50 most downregulated genes and were also included the genes involved in fatty acid metabolism and oxidative stress. This analysis was not possible to perform in Control vs CV + M due to the reduced number of identified DEGs. Table 1 displays the identified significant DEGs for comparisons Control *vs* CV and Control *vs* CV + R, its respective functional description, and Log2 FC.

**Table 1:** Identified DEGs by functional analysis with Cytoscape software for comparisons Control *vs* CV and Control *vs* CV + R, its respective functional description and Log2 FC.

Gene	Functional description	Log2 FC^1^ Control *vs* CV	Log2 FC^1^ Control *vs* CV + R
*AGPAT2*	Phosphatidic acid biosynthetic process	− 5.25	–
*ACSM2B*	Acyl-CoA metabolic process; Fatty acid biosynthetic process	− 4.71	–
*APOA1*	Participates in the reverse transport of cholesterol from tissues to the liver for excretion by promoting cholesterol efflux from tissues	− 4.63	–
*APOE*	Lipid transport	− 4.58	–
*CNEP1R1*	Positive regulation of triglyceride biosynthetic process	− 3.99	–
*ACOX3*	Fatty acid β-oxidation using acyl-CoA oxidase; Lipid homeostasis	–	− 3.80
*NDUFAB1*	Carrier of the growing fatty acid chain in fatty acid biosynthesis	− 3.54	− 2.50
*PNPLA2*	Positive regulation of triglyceride catabolic process	− 3.01	–
*MLYCD*	Malonyl-CoA catabolic process; Positive regulation of fatty acid oxidation	− 2.74	− 3.56
*PLBD1*	Phospholipase activity	− 2.6	–
*CYP2E1*	Hydroxylates fatty acids specifically at the omega-1 position displaying the highest catalytic activity for saturated fatty acids	− 2.58	–
*TYSND1*	Catalyses the processing of PTS1-proteins involved in the peroxisomal β-oxidation of fatty acids	–	− 2.91
*ACBD4*	Binds medium- and long-chain acyl-CoA esters and may function as an intracellular carrier of acyl-CoA esters	–	− 2.44
*ECHS1*	Fatty acid β-oxidation	− 2.42	–
*SOD1*	Destroys radicals which are normally produced within the cells and which are toxic to biological systems; Negative regulation of cholesterol biosynthetic process	− 2.37	− 2.16
*DNAJC15*	Regulation of lipid metabolic process	–	− 2.25
*PPARD*	Fatty acid β-oxidation; Fatty acid metabolic process	− 2.19	− 2.54
*HSD17B8*	Participating in mitochondrial fatty acid biosynthesis	− 2.1	–
*PISD*	Catalyses the formation of phosphatidylethanolamine (PtdEtn) from phosphatidylserine (PtdSer). Plays a central role in phospholipid metabolism	− 2.06	–
*ACADVL*	Fatty acid β-oxidation using acyl-CoA dehydrogenase; Negative regulation of fatty acid biosynthetic process; Regulation of cholesterol metabolic process	− 2.01	− 2.05
*CHPT1*	Diacylglycerol cholinephosphotransferase activity	− 1.9	–
*ECHDC2*	Fatty acid β-oxidation	− 1.85	–
*PEX5*	Binds to the C-terminal PTS1-type tripeptide peroxisomal targeting signal (SKL-type) and plays an essential role in peroxisomal protein import	–	− 1.83
*GPX4*	Plays a key role in protecting cells from oxidative damage by preventing membrane lipid peroxidation	− 1.83	− 1.76
*ACADL*	Catalyse the first step of mitochondrial fatty acid β-oxidation, an aerobic process breaking down fatty acids into acetyl-CoA and allowing the production of energy from fats	− 1.82	− 1.82
*ACOT8*	Catalyses the hydrolysis of acyl-CoAs into free fatty acids and coenzyme A (CoASH), regulating their respective intracellular levels	− 1.60	− 1.82
*LPCAT3*	Regulation of cholesterol biosynthetic process	–	− 1.63
*SIRT3*	Contributes to the regulation of cellular energy metabolism	–	− 1.62
*PEX7*	Binds to the N-terminal PTS2-type peroxisomal targeting signal and plays an essential role in peroxisomal protein import	–	− 1.58
*ACADS*	Catalyse the first step of mitochondrial fatty acid β-oxidation	–	− 1.57
*CPT1B*	Long-chain fatty acid transport; Fatty acid metabolic process	–	− 1.50
*TREX1*	Regulation of fatty acid metabolic process; Regulation of lipid biosynthetic process	–	− 1.50
*PCCA*	A mitochondrial enzyme involved in the catabolism of odd chain fatty acids, branched-chain amino acids isoleucine, threonine, methionine, and valine and other metabolites	–	− 1.48
*CIDEA*	Binds to lipid droplets and regulates their enlargement, thereby restricting lipolysis and favoring storage	− 1.48	–
*PPP2R5A*	Negative regulation of lipid kinase activity	− 1.46	–
*PCCB*	A mitochondrial enzyme involved in the catabolism of odd chain fatty acids, branched-chain amino acids isoleucine, threonine, methionine, and valine and other metabolites	− 1.35	–
*HADH*	Mitochondrial fatty acid β-oxidation enzyme that catalyses the third step of the β-oxidation cycle for medium and short-chain 3-hydroxy fatty acyl-CoAs (C4 to C10)	− 1.34	–
*TECR*	Very-long-chain enoyl-CoA reductase activity	− 1.34	–
*PHYH*	Catalyse de hydroxylation of methyl-branched fatty acids	–	− 1.33
*SOD2*	Destroys superoxide anion radicals which are normally produced within the cells and which are toxic to biological systems	− 1.34	− 1.26
*ACSS2*	Lipid biosynthetic process	− 1.09	–
*APOD*	Lipid metabolic process; Lipid transport	1.19	–
*HADHA*	Catalyses the last three of the four reactions of the mitochondrial β-oxidation pathway	1.21	–
*BRCA1*	Inhibits lipid synthesis by binding to inactive phosphorylated ACACA	1.29	–
*MGST2*	Glutathione biosynthetic process	1.42	–
*LPL*	Plays an important role in lipid clearance from the bloodstream, lipid utilisation and storage	1.48	–
*C3*	Adipogenic hormone that stimulates triglyceride (TG) synthesis and glucose transport in adipocytes, regulating fat storage and playing a role in post-prandial TG clearance. Appears to stimulate TG synthesis via activation of the PLC, MAPK and AKT signalling pathways	1.8	–
*RPS21*	Structural constituent of ribosome	–	1.55
*RPL39*	Structural constituent of ribosome	–	1.58
*RPS12*	Structural constituent of ribosome	–	1.60
*RPL35*	Structural constituent of ribosome	–	1.68
*NLRP3*	Inflammatory response	–	1.94
*SCD*	Unsaturated fatty acid biosynthetic process; Plays an important role in lipid biosynthesis. Plays an important role in regulating the expression of genes that are involved in lipogenesis; Contributes to the biosynthesis of membrane phospholipids	2.76	2.22
*CES1*	Cellular response to cholesterol; Negative regulation of cholesterol storage	4.21	–

^1^Log2 FC > 1—upregulated in Control group; Log2 FC < 1—downregulated in Control group.

**Figure 5:**
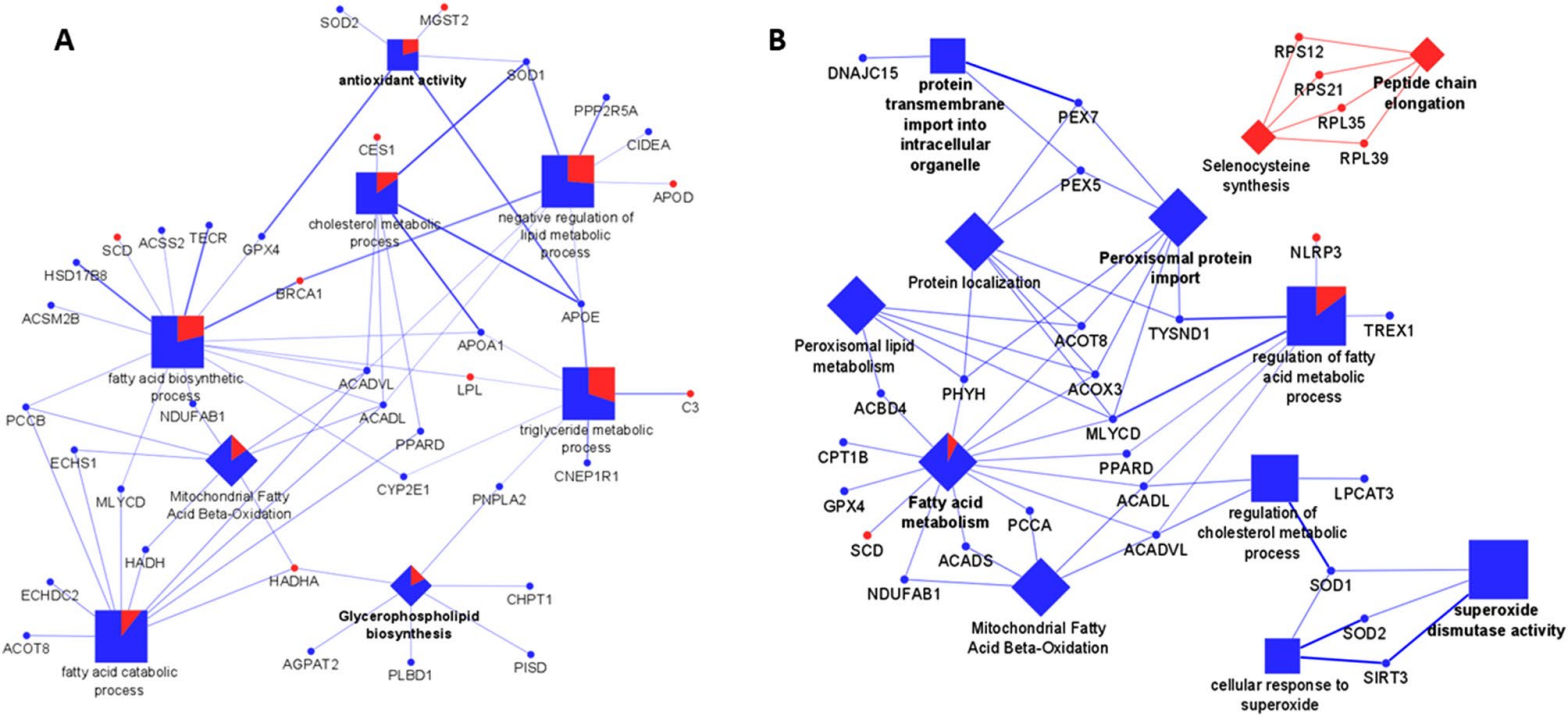
Functional analysis performed with Cytoscape software using the 50 most up and 50 most downregulated genes and genes of interest for (**A**): Control vs CV comparison and (**B**): Control vs CV + R comparison. The significant DEGs and the interactions with their related pathways and biological processes are presented in the figure. Legend: squares = biological processes; diamonds = reactome pathways; circles = DEGs; shape size = according to the p-value of the term in its own group; red colour = upregulated in Control; blue colour = downregulated in Control.

In Control *vs* CV (Fig. 5A), the majority of the DEGs are downregulated in the Control group and consequently, upregulated in CV and are marked as blue circles. Only the following DEGs: *SCD*, *BRCA1*, *CES1*, *MGST2*, *APOD*, *LPL*, *C3*, and *HADHA* are upregulated in Control and are marked as red circles. In this comparison, DEGs are associated to the following biological processes (squares): “Fatty acid catabolic process” (*P* = 1.3E-08), “Fatty acid biosynthetic process” (*P* = 4.9E−13), “Cholesterol metabolic process” (*P* = 5.4E-04), “Negative regulation of lipid metabolic process (*P* = 1.9E-06), “Triglyceride metabolic process (*P* = 8.8E-05), and “Antioxidant activity” (*P* = 0.008), and are associated to the following pathways (diamonds): “Mitochondrial fatty acid β-oxidation” (*P* = 2.4E-08), and “Glycerophospholipid biosynthesis” (*P* = 0.002).

Regarding Control *vs* CV + R (Fig. 5B), it was observed that all DEGs associated with “Peptide chain elongation” (*P* = 0.03) and “Selenocysteine synthesis “ (*P* = 0.02) pathways are upregulated in Control (red diamonds). For all other identified biological processes and pathways, the majority of the DEGs are downregulated in the Control group (blue circles), with the exception of *SCD* and *NLRP3* (red circles). The remaining biological processes (squares) identified are: “Protein transmembrane import into intracellular organelle” (*P* = 0.02), “Regulation of fatty acid metabolic process” (*P* = 1.8E-05), “Regulation of cholesterol metabolic process” (*P* = 6.9E-04), “Superoxide dismutase activity” (*P* = 4.6E-04), and “Cellular response to superoxide” (*P* = 0.009). The identified pathways (diamonds) are: “Fatty acid metabolism” (*P* = 3.3E−12), “Peroxisomal lipid metabolism” (*P* = 6.6E-06), “Protein localization” (*P* = 3.6E-04), “Peroxisomal protein import” (*P* = 5.4E-07), and “Mitochondrial fatty acid β-oxidation” (*P* = 2.3E-05).

In the comparison Control *vs* CV + M, three DEGs were identified. The ncRNA gene *MIR10390* and the protein-coding gene *LMO7* are upregulated in Control, while the protein-coding gene *IGFN1* is upregulated in the CV + M group.

### Proteomics analysis

A total of 1329 protein identifications were obtained in the muscle of finishing pigs following the LC–MS analysis (Supplementary file 2). The following sections describe the differentially abundant proteins between experimental groups (CV, CV + R, CV + M) and control.

### Control vs CV

21 proteins were differentially abundant between CV and control groups (Supplementary Table S2). The former had 11 proteins with higher abundance. These proteins included acyl-CoA binding domain containing 7 (ACBD7), and ribosomal protein S25 (RPS25), with fatty-acyl-CoA binding (GO:0000062), and structural constituent of ribosome (GO:0003735) molecular functions. It also had a higher abundance of contractile apparatus proteins including troponin T (tnnt3), and myosin light-chain 1 (MYL1). Conversely, control had higher abundance of other structural muscle proteins, myosin-2 (MYH2), and ACTN1. This group had also higher abundance of mRNA metabolism proteins, including 2-iminopropanoate deaminase (RIDA), and ribonucleoprotein D (HNRNPD) with mRNA catabolic process (GO:0006402), and positive regulation of translation (GO:0045727) biological process annotations, respectively.

### Control vs CV + R

This comparison has yielded 18 differentially abundant proteins, with 5 of them being highly abundant in CV + R (Supplementary Table S3). This latter group of proteins had heterogeneous biological process annotations including respiratory electron transport chain (GO:0022904, Rieske domain-containing protein -UQCRFS1), and fatty acid β-oxidation (GO:0006635, hydroxysteroid 17-β dehydrogenase 10—HSD17B10). In turn, control increased the abundance of several myosin proteins (MYH1, MYH2, MYH4 and MYH7), which are involved in muscle contraction/motor activity (GO:0003774). It also increased the abundance of ATP binding (GO:0006883) proteins such as sodium/potassium-transporting ATPase subunit alpha (ATP1A3), and creatine kinase (CKMT1), with sodium/potassium homeostasis (GO:0006883, GO:0030007), and phosphocreatine biosynthetic process (GO:0046314) biological process annotations, respectively.

### Control vs CV + M

This comparison had the lowest number of differentially abundant proteins, with 5 and 7 highly abundant proteins in CV + M and control, respectively (Supplementary Table S4). The former group had higher abundance of ribosome (RPS17), and SERPIN domain-containing (SERPINA3-2) proteins which participate in translation (GO:0006412), and negative regulation of endopeptidase activity (GO:0010951), respectively. It also increased the abundance of SPARC, a protein that regulates cell growth (anatomical structure development, GO:0048856). Control increased the abundance of myosin-binding protein H (MYBPH), and ACTN1, both being contractile apparatus proteins. In addition, they also increased the abundance of NADH dehydrogenase ubiquinone iron-sulphur protein 3 (NDUFS3), which partakes in mitochondrial electron transport (GO:0006120), and creatine kinase (CKMT1).

### Data integration

A supervised analysis was conducted using the DIABLO procedure to detect multi-omics signatures that distinguish the experimental groups. A threshold of 0.7 was used to illustrate significant correlations between genes and proteins, as seen in the circosplots of Figure S3.

### Control vs CV

In this comparison, the two datasets presented a high correlation of 0.94 (Figure S4A). The correlation circle plot obtained for this comparison demonstrates three separate clusters of genes and proteins that positively correlate with each other (Figure S5A). These are depicted in the circosplot obtained in Figure S3A, which shows a high number of positive correlations. Two contractile apparatus proteins, ACTN1 (I3LLY3) and MYBPH (I3LIE7), actin and myosin-binding proteins, are positively correlated with genes including *NCAPD3* (participates in cell division), *SCD* (participates in lipid metabolism), and *ZNF879* (regulates transcription).

Histone H4 (P62802) is positively correlated with *FANCD2,* and *NCAPD3*, two genes with maintenance of chromosomal stability and histone binding functions, respectively.

The ribosomal protein RPS25 (F2Z5G8), and the collagen-binding SPARC protein (P20112) were negatively correlated with the *PAPPA2* gene. The latter is involved in proteolysis and regulates cell growth.

### Control vs CV + R

This comparison yielded a high dataset correlation of 0.95, similarly to the CV vs control comparison (Figure S4B). As seen in Figure S5B and Figure S3B, there is a negative correlation of a cluster of genes with a particular protein, CAMK2A (I3LNG5). These genes include *BAX* (involved in apoptosis), *DDA1* (positively regulates protein catabolism), and *MAD2L2* (negatively regulates transcription). The aforementioned protein is a kinase that activates transcription in developing neurons. Interestingly, another protein (HNRNPD—F1RVC9), which regulates gene expression, is positively correlated with these genes. Both of these proteins are highly abundant in control.

In turn, three myosin proteins (MYH1, MYH4, MYH7), NEB (actin binding), and CRYAB (muscle development) are positively correlated with lipid metabolism genes such as *CIDEC* (lipid droplet organisation/biogenesis), and *SCD* (phospholipid, cholesterol and triglyceride synthesis). These genes and proteins were all highly abundant in the control group compared to CV + R.

### CV + M vs control

This comparison yielded the lowest number of differentially expressed genes and abundant proteins, achieving the lowest correlation between the two datasets (Figure S4C). As seen in the correlation circle plot (Figure S5C) and circosplot (Figure S3C), there is a positive correlation between the *IGFN1* gene and three proteins: SPARC (P20112), SERPIN domain-containing protein (F1SCC6), and TMEM263 (A0A287B182) proteins. Likewise, there is another positive correlation between two genes (*LMO7* and *mir10390*) and four proteins: ACTN1 (I3LLY3), NDUFS3 (A0A286ZNN4), CAMK2A (I3LNG5), and TUBA4A (F2Z5S8).

## Discussion

With the new challenges imposed on the livestock industry, it is imperative to find environmentally sustainable alternatives to conventional feedstuffs. Among the former, *C. vulgaris* takes a prominent place due to their nutritional and production characteristics^2,12^. In a companion paper^34^, we evaluated, for the first time, the effect of a high dietary incorporation level of *C. vulgaris* (5%), alone or supplemented with two exogenous CAZyme mixtures to disrupt the microalga cell wall in finishing pig diets. We found that this high dietary incorporation of *C. vulgaris* did not lead to significant differences in productive performance and meat quality but allowed an increase in muscle deposition of carotenoids and *n*-3 PUFA. In addition, we also observed that the supplementation with CAZymes did not promote significant changes in most studied parameters. To the best of our knowledge, the effect of the dietary incorporation of *C. vulgaris* in pig muscle transcriptome and proteome has never been assessed. Herein, we studied for the first time the effect of a high dietary incorporation level of *C. vulgaris* (5%), alone or supplemented with two exogenous CAZyme mixtures, on pig muscle transcriptome and proteome in an integrated approach.

Regarding the differential gene expression among groups, we found a decreasing number of significant DEGs identified from Control *vs* CV, Control *vs* CV + R and lastly Control *vs* CV + M, with a clear discrimination between Control group and CV and CV + R groups and a reduced discrimination between Control and CV + M. In fact, only three genes presented differential expression in the latter comparison. Thus, we verified that the dietary incorporation of 5% *C. vulgaris* alone and supplemented with 0.005% of Rovabio® Excel AP promoted a high modification on finishing pig muscle transcriptome, whereas the supplementation with 0.01% of the four-CAZyme mixture had incipient effects on the muscle transcriptome compared to controls. This mixture proved to be effective in in vitro studies on *C. vulgaris* cell wall disruption, leading to an increase in the bioaccessibility of its nutrients such as proteins, *n*-3 PUFA and carotenoids^23^. Thus, we hypothesised that the animals fed with diets containing *C. vulgaris* alone and supplemented with Rovabio® Excel AP were less able to digest *C. vulgaris* cell wall when compared to pigs fed the diet supplemented with four-CAZyme mixture, which allowed the digestion of the cell wall polysaccharide matrix into structurally simpler oligosaccharides^23^. Therefore, without an efficient disruption of *C. vulgaris* cell wall, the digestion of this biomass in the gastro-intestinal tract of pigs is compromised, blocking the access to valuable intracellular nutrients^36–38^. Although these dietary treatments did not influence zootechnical performance and meat quality traits in finishing pigs^34^, they led to significant changes in the muscle transcriptomic profile, triggering the expression of genes involved in energy homeostasis biological processes, such as lipid metabolism and mitochondrial function, functioning as a compensatory system. The study by Skugor et al*.*^39^ supports our premise. These authors tested the effects of long-term feeding of rapeseed meal on skeletal muscle transcriptome, production efficiency and meat quality traits in growing-finishing pigs. The authors observed that a dietary incorporation of 20% rapeseed promoted the reduction of growth performance and the upregulation of genes involved in lipid metabolism and mitochondrial function in order to keep the energy homeostasis. The authors discuss these results as a consequence of a higher lower-degradable fibre content in the rapeseed diet in comparison with the Control group, which reduces the availability of energy and nutrients^39^. In our study, the dietary incorporation of 5% of *C. vulgaris* alone or supplemented with 0.005% of Rovabio® Excel AP promoted the activation of similar pathways and biological processes.

Our bioinformatic analysis with Cytoscape and DIABLO tools identified common significant DEGs: *SCD* in the comparison Control *vs* CV and *SCD*, *ACOT8* and *SIRT3* in the comparison Control *vs* CV + R.

The gene *SCD*, downregulated in the CV and CV + R groups compared with Control, is involved in fatty acid biosynthetic process. The *SCD* encodes the stearoyl-CoA desaturase, a rate-limiting enzyme that catalyses the synthesis of monounsaturated fatty acids, such as 16:1 and 18:1, from 16:0 and 18:0 that are either synthesised de novo or derived from the diet^40,41^. *SCD* expression is regulated by several mechanisms: (A) upregulation through an high glucose-uptake via sterol regulatory element-binding protein (SREBP-1c) activation dependently of insulin^41,42^, (B) via carbohydrate response element binding protein (ChREBP) activation independently of insulin^43^ or (C) downregulation through an high dietary PUFA intake via the mechanism proposed by Corominas et al*.*^44^. The authors suggest that *ChREBP* gene function is supressed by PUFA high intake levels, inhibiting lipogenic genes, including *SCD*. According to our results, we conclude that the downregulation of *SCD* gene in the CV and CV + R groups is not due to a high consumption of PUFA, since this was not observed in the CV + M diet and there were no differences in *ChREBP* gene expression. Indeed, the content of PUFAs in the CV incorporated diets was likely not high enough to promote the downregulation of *SCD* gene. Instead, we propose that the inefficient *C. vulgaris* cell wall disruption in the gastrointestinal tract of animals fed with CV and CV + R diets may have limited the normal glucose-uptake, promoting a downregulation of the *SCD* gene via SREBP-1c and ChREBP. Although there were no differences in mRNA expression of SREBP-1c and ChREBP, a low glucose-uptake may have implications for the post-translational processing of these transcription factors, decreasing its activation, thus leading to lower expression of the *SCD* gene^43,45^. Nonetheless, it must be stressed that the downregulation of *SCD* gene in muscle transcriptome of pigs had no repercussions on the composition of muscle monounsaturated fatty acids profile^34^.

Regarding Control *vs* CV + R, the *SIRT3* and *ACOT8* genes, involved in superoxide dismutase activity and peroxisomal lipid metabolism, respectively, are upregulated in the CV + R group. *SIRT3* gene encodes a member of the sirtuin family of class III NAD^+^-dependent histone deacetylases^46^. This enzyme is localised primarily in mitochondria and has been shown to deacetylate and thereby regulate several mitochondrial targets, including acetyl-CoA synthase 2 and glutamate dehydrogenase. It has been suggested that *SIRT3* is also closely involved in energy homeostasis and, recently, it was demonstrated that *SIRT3* plays an important role in hepatic lipid metabolism^47^. Moreover, this enzyme is involved in the control of the levels of reactive oxygen species (ROS), helping to maintain a normal cellular redox state^47^. *SIRT3* expression has been observed to change under a number of different stresses in multiple tissues and model systems^48^. It is well established, in mice model, that caloric restriction and nutrient deprivation promote an increase of *SIRT3* mRNA in different tissues, including skeletal muscle^49,50^. One of the mechanisms that explains the relation between the expression of the gene *SIRT3* and nutrient deprivation relies on its involvement in superoxide dismutase activity. ROS have been shown to increase in mice skeletal muscle as a consequence of fasting and as SIRT3 has been shown to play a role in ROS scavenging, increasing SIRT3 protein may be an adaptation to handling a prolonged period of increased ROS production^51^. The increased expression of *SIRT3* and consequent activation of SIRT3 protein will modify downstream pathways in order to control the oxidative stress and keep energy homeostasis^52^. Therefore, in our study, the increased expression of *SIRT3* in *longissimus lumborum* muscle of animals fed with CV + R diet could be a physiological response to a lower nutrient intake caused by the decrease in the bioavailability of nutrients provided by *C. vulgaris* due to the non-efficient disruption of its cell wall, to maintain a proper mitochondrial functioning. Interestingly, this gene is not upregulated in the CV group compared with Control.

The *ACOT8* encodes the Type-II acyl-coenzyme A thioesterase 8 (ACOT8) that catalyses the hydrolysis of acyl-CoA to the free non-esterified fatty acid and coenzyme A (CoASH), providing the potential to regulate intracellular levels of acyl-CoA, free fatty acids and CoASH^53–55^. The acyl-coenzyme A thioesterases are localised in almost all cellular compartments, whereas the location of ACOT8 is peroxisomal^56^. According to Kirkby et al*.*^56^, the regulation of ACOT gene expression can be modulated via peroxisome proliferator-activated receptors (PPARs). These are ligand activated transcription factors of which three isotypes exist: alpha (PPARɑ), delta (PPARδ) and gamma (PPARγ), being the *ACOT8* expression upregulated by PPARs^56,57^. Westin et al*.*^58^ conducted a study with the aim of unveiling the function of ACOTs in mice model. The authors claim that fasting triggers an upregulation of the peroxisomal ACOT8 gene via activation of PPARs leading to the hydrolysis of acyl-CoA into medium-chain dicarboxylic acids and CoASH. Interestingly, in our study we observed an upregulation of *PPARδ* gene in CV and CV + R group comparing to the Controls similarly to the *ACOT8* gene. Therefore, we hypothesise that the nutrient deprivation caused by the inefficient *C. vulgaris* cell wall disruption in these experimental groups promoted an upregulation of *PPARδ* gene. This in turn had a positive effect on the expression of *ACOT8* gene with the aim of regulating the levels of acyl-CoA, free fatty acids and CoASH in order to prepare the muscle cell to a state of higher nutritional deficiency, since the overexpression of *ACOT8* gene is associated with lipid accumulation and possibly a more adipogenic phenotype^59^. However, the relation between nutrient deprivation and upregulation of *PPARδ* and *ACOT8* genes needs further investigation.

Regarding proteomics, particularly the Control *vs* CV comparison, the results demonstrate a differentiated muscle protein metabolism between both groups, since these had differentially abundant muscle contractile apparatus proteins on both sides (MYL1, TNNT3, MYH2, MYBPH, ACTN1). These proteins have been previously related with muscle development; however, this has not been translated into higher live weight gains in this study. Two of these genes (*MYBPH* and *ACTN1*) were positively correlated with *NCAPD3*, *SCD* and *ZNF879* genes, all of which were more abundant/highly expressed in Control animals. *NCAPD3* has been reported to be highly abundant in the muscle of Berkshire pigs with low intramuscular fat^60^, whereas *ZNF879* codes for a zinc finger protein that regulates transcription^61^. In the present study, there were no differences between groups regarding IMF deposition^34^. However, muscle proteins could be related with these genes due to the inverse relationship of IMF deposition and muscle development and the higher transcription/translation rates associated with higher muscle protein synthesis. Interestingly, the *PAPPA2* gene (highly expressed in control) is negatively correlated with two proteins (highly abundant in CV): RPS25 and SPARC. The former cleaves IGFBP5, which has been reported to potentiate the action of IGF1^62^ and might be associated with fat deposition in pigs^63^. In turn, the two proteins are related with protein synthesis and regulation of collagen synthesis and modification^64^, respectively. Therefore, we hypothesise that this negative correlation could reflect the different rates of extracellular matrix (higher in CV) and fatty acid (lower in Control) accumulation. Madeira et al*.*^65^ have found that pigs fed with reduced protein diets (13% as fed) have higher intramuscular fat (IMF) accumulation. Accordingly, we found that diets with high levels of CV have lower crude protein digestibility in weaned piglets^38^. In addition, CV accumulated significantly higher levels of PUFA (18:3*n*-3, 18:4*n*-3 and 20:5*n*-3) in the muscle^34^, supporting the aforementioned hypothesis.

The Control *vs* CV + R comparison yielded similar results. The control group had higher abundance of several myosins (MYH1, MYH2, MYH4 and MYH7), nebulin (NEB—an actin binding protein), and reticulocalbin 3 (RCBN3) that participates in collagen biosynthesis. Instead of reflecting higher muscle development, this could reflect the higher nutrient availability of the control diet. The CV + R diet had detrimental effects in the digestibility of several nutrient fractions, including crude protein, when fed to weaned piglets^38^. The same could occur in the finishing pigs of this study, thereby comparatively increasing protein synthesis rates and overall anabolism in control piglets. Indeed, these also had increased abundance of creatine kinase (CKMT1), which generates phosphocreatine from creatine and ATP, a metabolite with a central role in muscle energy storage^66^. The higher nutrient availability caused by standard feed supplementation with non-starch polysaccharide enzymes has also been responsible for an increased abundance of proteins related to protein synthesis and muscle differentiation, in the muscle of finishing pigs^67^. The Rovabio® mixture is also composed of such enzymes (e.g. xylanase, β-glucanase), however, because it is not specific to the recalcitrant cell wall polysaccharides of *C. vulgaris*, it may not improve its nutrient availability as mentioned above. Accordingly, the CV + R group had increased abundance of Rieske domain-containing protein (UQCRFS1), which is involved in glucose catabolism^68^ and mitochondrial acyl-CoA dehydrogenase (ACADS), involved in the β-oxidation of short-chain fatty acids^69^. Both differences support higher endogenous nutrient consumption to compensate for reduced nutrient digestibility in the CV + R group compared to control. The integrative analysis carried out using DIABLO has yielded similar results to the Control *vs* CV comparison. Indeed, we found that myosin proteins (MYH1, MYH4, MYH7), NEB and CRYAB were positively correlated to *SCD* and *CIDEC* genes, all highly abundant/expressed in control. Indeed, this reinforces the pattern by which Control pigs had increased protein synthesis rates in the muscle, while simultaneously increasing the expression of *SCD* and *CIDEC* genes. These last two genes are upregulated in response to higher glucose uptake in Control. In addition, a cluster of genes related to apoptosis (*BAX*), protein catabolism (*DDA1*) and negative regulation of transcription (*MAD2L2*) were negatively correlated with CAMK2A. These genes were upregulated in CV + R and the protein in Control. The latter is necessary to maintain muscle calcium homeostasis^70^, of particular importance for muscle contraction since these pigs also had increased abundance of several contractile apparatus proteins. The genes reflect comparatively increased catabolic rates of muscle protein in CV + R pigs, as a putative compensatory mechanism for reduced crude protein digestibility.

Lastly, the Control *vs* CV + M comparison depicted the lowest number of differentially abundant proteins and expressed genes. The lack of differences found may reflect the ability of the four CAZyme mix to improve nutrient availability compared CV and CV + R group, maintaining Control-like homeostasis. Control pigs had increased abundance of CKMT1 and NDUFS3. The former is a kinase already mentioned to take part in phosphocreatine synthesis. The latter is a NADH dehydrogenase involved in the assembly of mitochondrial respiratory chain complex I^71^, which follows the trend for upregulation of energy-generating pathways in control pigs. Conversely, CV + M pigs increased the abundance of the SPARC protein, involved in collagen synthesis^72^. Moreover, it increased the abundance of one myosin (MYL1) and a ribosomal protein (RPS17). Altogether, these results suggest increased muscle protein synthesis. These have not resulted however, in a higher muscle development and consequently weight gain^34^. Therefore, these results likely demonstrate higher anabolic rates in CV + M compared to the remaining *C. vulgaris* fed pigs. The DIABLO integration yielded two positive correlations between different genes and proteins. *LMO7*, a gene that has been related with muscle development and ham weight in Duroc pigs^73^, was highly expressed in the Control group. It was positively correlated with actinin-1 (ACTN1), NDUFS3, CAMK2A and TUBA4A. These proteins have been mentioned to reflect higher energy production/nutrient utilisation rates of the tissue from pigs fed a control diet, coherent with the higher nutrient availability mentioned in an analogous piglet trial^38^.

**Figure 6:**
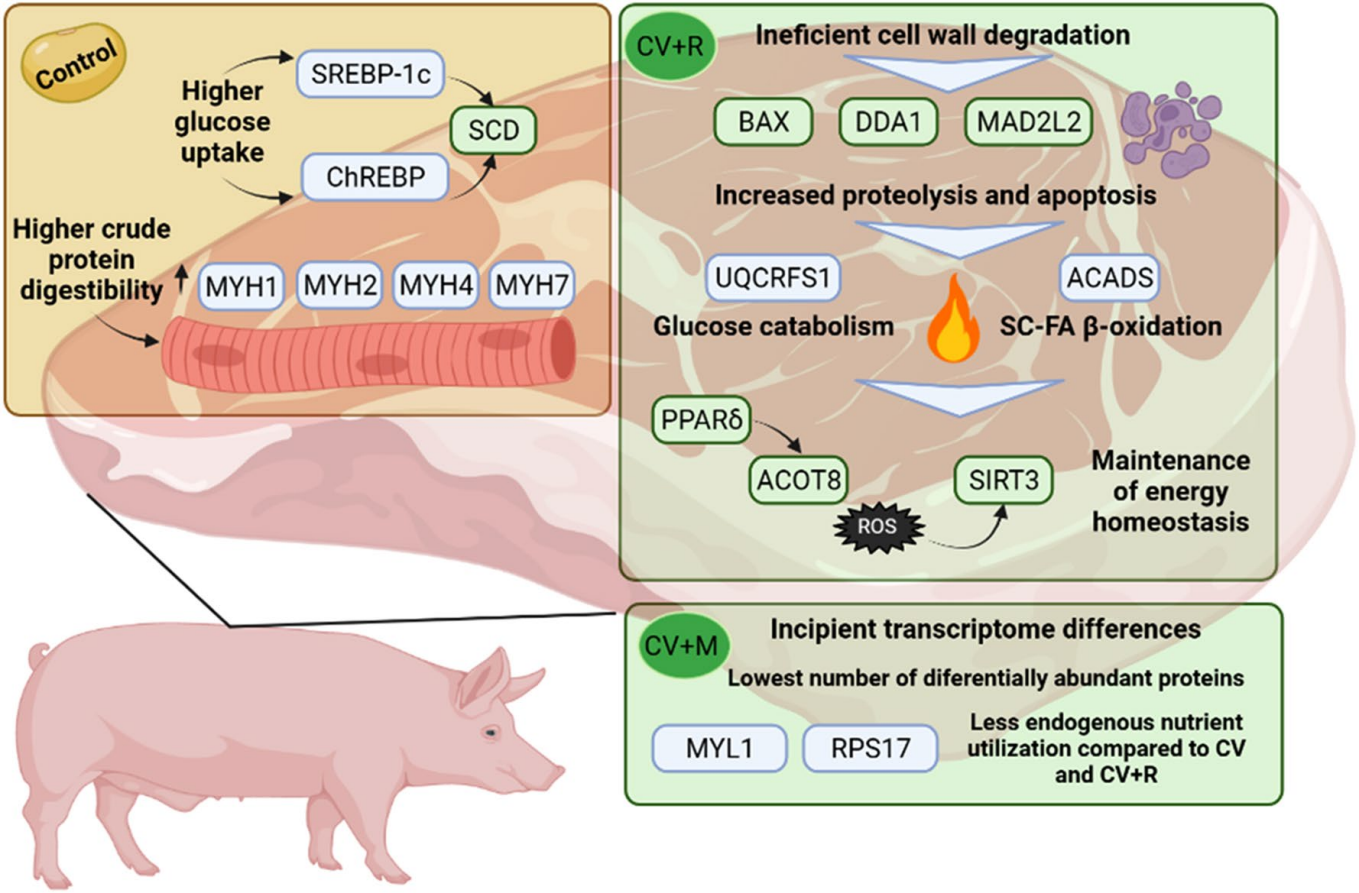
A summary of the main results obtained in the transcriptome and proteome of the *longissimus lumborum* muscle of finishing pigs. Figure created using Biorender.Com.

The bioinformatic analysis performed in this experiment allowed us to narrow down the most interesting and informative genes and proteins, as well as to reveal multi-omics patterns that distinguish our experimental groups. It is, to our knowledge, the first time that such an approach was conducted in this context. The main results that have emerged are summarized in Fig. 6. Overall, control pigs had increased abundance of proteins related to muscle contractile apparatus, possibly related to increased availability of dietary protein. In turn, CV and CV + R pigs demonstrated signs of inefficient or absent cell wall degradation, since they upregulated proteolytic and apoptotic genes. In addition, they had increased abundance proteins involved in glucose and short-chain FA catabolism, highlighting the importance of the mobilization of body tissues as energy sources. The CV + M group had the lowest number of significantly identified genes and proteins, demonstrating that the mix was able to mitigate the detrimental effects of *C. vulgaris* inclusion in feeds and the adverse consequences in pig muscle metabolism.

## Conclusion

This study describes, for the first time, the effect of dietary *C. vulgaris* and CAZyme supplementation on the muscle transcriptome and proteome of finishing pigs using an integrated approach. We have demonstrated that the four-CAZyme mix, designed to degrade the microalgal cell wall, is able to mitigate the otherwise detrimental effects that were detected in the muscle of CV and CV + R pigs. These impacts include increased proteolysis, apoptosis, glucose and, fatty acid catabolism. We have thus integrated the information obtained from the two Omics platforms in a data-driven approach, demonstrating their potential and providing patterns that clearly distinguish our experimental groups. In the future, extending this analysis to include metabolomics would allow an even more robust and in-depth analysis. For data integration purposes, increasing the number of biological replicates would also improve the analysis, which is often not easy to accomplish in farm animal trials due to financial constraints. Performing this integrative analysis in the liver would help provide a systemic overview of the metabolism as influenced by the diet.

## Material and methods

### Animal trial and experimental diets

Animal trial was done at the Unidade de Investigação em Produção Animal of the Instituto Nacional de Investigação Agrária e Veterinária (UEISPA-INIAV, Santarém, Portugal) facilities. All in vivo procedures were approved by the Ethics Commission of the Centro de Investigação Interdisciplinar em Sanidade Animal/Faculdade de Medicina Veterinária (CIISA/FMV) and the Animal Care Committee of the National Veterinary Authority (Direcção-Geral de Alimentação e Veterinária, Portugal), in line with the European Union guidelines (2010/63/EU Directive) on animal experimentation and the ARRIVE guidelines 2.0 (https://arriveguidelines.org/arrive-guidelines). All the staff members involved in the animal experiment are licensed from the Portuguese Veterinary Services.

The in vivo trial has been described in detail in a companion paper^34^. Briefly, forty crossbred entire male pigs, (Large White × Landrace) sows × Pietrain boars, with an initial body weight of 59.1 ± 5.69 kg were randomly allocated into 10 pens, each with 4 pigs. Four experimental diets were randomly attributed to pigs within each pen. Animals were individually fed, allowing each to receive a different diet, and the pig was the experimental unit. The experimental diets were: Control, a cereal and soybean meal-based diet; CV, control diet with 5% *C. vulgaris* provided by Allmicroalgae (Natural Products, Portugal); CV + R, CV diet supplemented with 0.005% of Rovabio® Excel AP (Adisseo, Antony, France); and CV + M, CV diet supplemented with 0.01% of the preselected four-CAZyme mixture^23^. The CV + R group was included in order to compare the response of the well-known and documented enzyme mix (Rovabio® Excel AP) with the previously mentioned experimental mix. The chemical analysis and composition of these diets has been described previously by Coelho et al*.*^34^.

### Animals slaughter and sample collection

Pigs were slaughtered at the Unidade de Investigação em Produção Animal experimental slaughterhouse (Santarém, Portugal), with a body weight of 101 ± 1.9 kg. Samples for transcriptomic and proteomic analysis were collected from the centre of *longissimus lumborum* muscle. For transcriptomics, samples were rinsed with sterile RNAse-free cold saline solution, cut into small pieces, stabilised with DNA/RNA Shield® reagent (Zymo Research, Irvine, CA, USA) and stored at − 20 °C until RNA extraction. For the proteomic analysis, samples were snap-frozen in liquid Nitrogen, and kept at − 80 °C until further analysis.

### Total RNA extraction

Total muscle RNA was isolated from muscle samples of six pigs of each experimental group (n = 6). The RNA was extracted and purified using the standard RNA extraction method with TRIzol (Invitrogen, Carlsbad, CA, USA) and the commercial RNeasy mini kit (Qiagen, Hilden, Germany), respectively. Then, RNA samples were subjected to a DNA digestion treatment with DNAse I (Qiagen, Hilden, Germany). All procedures followed the manufacturer’s instructions, as described^74^. The quantification of RNA was carried out using a spectrophotometer (Nanodrop ND-2000c, NanoDrop, Thermo Fisher Scientific, Willmington, DE, USA). The A260/280 ratios ranged between 1.9 and 2.1. The purified total RNA samples were subsequently checked for quality on Fragment Analyzer (Agilent Technologies, Santa Clara, CA, USA), using High Sensitivity RNA Analysis Kit (Agilent Technologies, Santa Clara, CA, USA). The samples were stored at – 80 °C until further procedures.

### cDNA synthesis, library preparation and sequencing

Full-length cDNA was obtained from 1 μg of RNA, with the cDNA Library Construction Kit (Clontech, San Jose, CA, USA), according to the manufacturer's instructions. RNA-libraries were prepared using the Smart-seq2 protocol adapted from Macaulay et al*.*^75^. Illumina libraries were performed using the protocol adapted from Baym et al*.*^76^. This procedure was performed and optimised by the Genomics Unit of the Instituto Gulbenkian Ciência (Oeiras, Portugal). Quantification and quality check of libraries were done using the Agilent Fragment Analyzer in combination with HS NGS Kit. Libraries were sequenced on an Illumina NextSeq500 Sequencer (Illumina, San Diego, CA, USA) using a 75 SE high throughput kit.

### RNA-Seq data analysis

After sequencing was completed, image data were outputted and transformed into raw reads and stored with a FASTQ format. Then, the raw data was processed. The quality control of raw reads was performed using the FastQC tool (http://www.bioinformatics.babraham.ac.uk/projects/fastqc/), which generates a report for each sample read set. After that, raw reads were trimmed using fastp tool^77^ with a Phred-quality threshold of 30 to eliminate Illumina adapters and bases with a quality Phred score lower than 30. A re-check of the quality of the resulting datasets of trimmed reads was performed after assuring that there were no problems or relevant biases with the data. The alignment of the high-quality reads with the *Sus scrofa* genome (Sus_scrofa.Sscrofa11.1.dna.toplevel) was made using hisat2 tool (http://daehwankimlab.github.io/hisat2/) with default parameters. Quality control of the alignment procedure was accessed with Qualimap tool (http://qualimap.conesalab.org/). As expected, the majority of reads aligned to exonic regions.

### Differentially expressed genes analysis

After raw reads processing and alignment with porcine reference genome, the gene expression value was quantified using the program FeatureCounts implemented in Subread software (version 1.5.1)^35^ as raw fragment counts. The identified genes were assessed for differential expression genes (DEGs) among experimental diets: Control *vs* CV, Control *vs* CV + R, and Control *vs* CV + M for a total of three comparisons. These comparisons were performed using DESeq2 (version 1.34.0) with an adjusted *p*-value or false discovery rate (FDR) threshold of 0.05 and a log2 fold-change (FC) threshold of − 1 and 1^78^. Default parameters in DESeq2 were used. For the comparison test, a multi-dimensional scaling analysis (MDS) plot was calculated to show the general relationship between the samples, a hierarchical clustering of treatment condition samples based on Pearson and Spearman correlations was performed and a volcano plot showing the relationship between FC and evidence of differential expression (-log FDR) was also protracted using the R language ggplots2 package (version 3.3.5).

### Functional enrichment analysis

The Cytoscape v3.8.2 software (Institute for Genomics and Bioinformatics, Graz University of Technology, Graz, Austria) was used for functional enrichment analysis on the 50 most up/downregulated genes of each comparison. This was performed using the ClueGO plug-in v2.5.8^79^, using the parameters described by Sirri et al*.*^31^. Briefly, the test was set with a right-sided hypergeometric distribution, and Bonferroni P-value correction was used. Minimum clustering was set at P ≤ 0.05 and minimum κ-score at 0.4. The biological process (BPs) ontology and REACTOME pathways were used as databases. Gene Ontology (GO) levels were set from 6 to 8, and the minimum number of genes/cluster was set at 5.

### LC–MS proteomics analysis

Procedures for proteomics analysis have been previously described^80^. Briefly, six samples were randomly chosen per experimental group (n = 6). They were processed in tubes containing lysing matrix A (MP Biomedicals, Irvine, CA, USA) and lysis buffer (100 mM Tris–HCl pH 8.5, 1% sodium deoxycholate (SDC), 10 mM tris (2-carboxyethyl) phosphine (TCEP), 40 mM chloroacetamide (CAA) and protease inhibitors. Protein homogenization was performed using the FastPrep-24 equipment (MP Biomedicals, Eschwege, Germany) at 6 m/s in 3 cycles of 30 s each, with intervals of 5 min at 4 °C. Protein extracts were then centrifuged for 5 min at 13,400 rpm and transferred into 1.5 mL low protein binding tubes. All extracts were incubated for 10 min at 95 °C under agitation (Thermomixer, Eppendorf, Hamburg, Germany), then sonicated for ten cycles of 30 s at 4 °C (Bioruptor, Diagenode, Liège, Belgium) and centrifuged again. The lysate was transferred onto a new 1.5 mL tube. Then, 100 µg of protein from each sample was processed for proteomics analysis. Enzymatic digestion was performed with trypsin/LysC (2 µg) overnight at 37 °C at 1000 rpm. Peptide concentration was measured by fluorescence.

Protein identification and quantitation were performed by nanoLC-MS/MS using an Ultimate 3000 liquid chromatography system coupled to a Q-Exactive Hybrid Quadrupole-Orbitrap mass spectrometer (Thermo Scientific, Bremen, Germany). Five hundred nanograms of peptides of each sample were loaded onto a trapping cartridge (Acclaim PepMap C18 100 Å, 5 mm × 300 µm i.d., 160,454, Thermo Scientific, Bremen, Germany) in a mobile phase of 2% ACN, 0.1% FA at 10 µL/min. After 3 min loading, the trap column was switched in-line to a 50 cm × 75 µm inner diameter EASYSpray column (ES803, PepMap RSLC, C18, 2 µm, Thermo Scientific, Bremen, Germany) at 250 nL/min. Separation was achieved by mixing A: 0.1% FA and B: 80% ACN, 0.1% FA with the following gradient: 5 min (2.5% B to 10% B), 120 min (10% B to 30% B), 20 min (30% B to 50% B), 5 min (50% B to 99% B), and 10 min (hold 99% B). The column was equilibrated with 2.5% B for 17 min. Data acquisition was controlled by Xcalibur 4.0 and Tune 2.9 software (Thermo Scientific, Bremen, Germany).

The mass spectrometer was operated in the data-dependent (dd) positive acquisition mode alternating between a full scan (m/z 380–1580) and subsequent HCD MS/MS of the 10 most intense peaks from a full scan (normalized collision energy of 27%). The ESI spray voltage was 1.9 kV. The global settings were as follows: use lock masses best (m/z 445.12003), lock mass injection Full MS and chromatographic peak width (FWHM) of 15 s. The full scan settings were as follows: 70 k resolution (m/z 200), AGC target 3 × 106, maximum injection time 120 ms; dd settings: minimum AGC target 8 × 103, intensity threshold 7.3 × 104, charge exclusion: unassigned, 1, 8, > 8, peptide match preferred, exclude isotopes on, and dynamic exclusion 45 s. The MS2 settings were as follows: microscans 1, resolution 35 k (m/z 200), AGC target 2 × 105, maximum injection time 110 ms, isolation window 2.0 m/z, isolation offset 0.0 m/z, dynamic first mass, and spectrum data type profile.

### Data treatment and analysis

Raw data were processed using the Proteome Discoverer 2.4.0.305 software (Thermo Scientific, Bremen, Germany). Protein identification analysis was performed with the data available in the UniProt protein sequence database for the *Sus scrofa* Proteome (2019_11) with 49,571 entries. The Sequest search node was considered with an ion mass tolerance of 10 ppm and 0.02 Da for precursor and fragment ions, respectively. Missed cleavage tolerance was set to two. Cysteine carbamidomethylation was defined as a fixed modification. The following variable modifications were considered: methionine oxidation, protein N-terminus acetylation, loss of methionine and Met-loss + Acetyl. Peptide confidence was set to high. The processing node Percolator was enabled with the following settings: maximum delta Cn 0.05; decoy database search target False Discovery Rate—FDR 1%; validation based on q-value. Protein-label-free quantitation was performed with the Minora feature detector node at the processing step. Precursor ion quantification was performing at the processing step with the following parameters: Peptides: unique plus razor; precursor abundance was based on intensity; normalization mode was based on the total peptide amount; the pairwise protein ratio calculation and hypothesis test were based on a t-test (background based).

For determination of differentially abundant proteins between experimental conditions, the following filters were considered following previously published protocols^23,24^: the number of unique peptides was set to 2 (minimum), the p-value set to < 0.05 and each protein had to be identified in at least half the samples. The protein fold change was set to > 1.5 (high abundance) and < 0.67 (low abundance). The MS proteomics data has been uploaded to the ProteomeXchange Consortium^81^ via the PRIDE^82^ partner repository with the dataset identifier PXD033814.

### Data integration analysis

Multi-omics data integration was performed using the Data Integration Analysis for Biomarker discovery using Latent cOmponents (DIABLO) procedure^83^ of the mixOmics package in the R Studio environment. Differentially expressed genes and abundant proteins of the Control vs CV, Control vs CV + R and Control vs CV + M comparisons were used as input. Novel genes (from transcriptomics data) were removed prior to analysis. Transcriptomic and proteomic data were centred and scaled. The supervised analysis was performed for each comparison using the *block.plsda* function, using the first two principal components and a full weighted design, to maximise the correlation between omics datasets. The workflow used for analysis is described at the mixOmics website https://mixomicsteam.github.io/Bookdown/diablo.html (accessed 06/04/2022).

## Supplementary Information

Supplementary Information 1.

Supplementary Information 2.

## Data Availability

The datasets generated during and/or analysed during the current study are available from the corresponding author on reasonable request. Proteomics raw data has been submitted in ProteomeXchange Consortium via the PRIDE partner repository with the dataset identifier PXD033814.

## References

[1] Ribeiro DM (2021). Quality traits and nutritional value of pork and poultry meat from animals fed with seaweeds. Foods.

[2] Madeira MS (2017). Microalgae as feed ingredients for livestock production and meat quality: A review. Livest. Sci..

[3] Fehlenberg V (2017). The role of soybean production as an underlying driver of deforestation in the South American Chaco. Glob. Environ. Chang..

[4] Morgan CA, Noble RC, Cocchi M, McCartney R (1992). Manipulation of the fatty acid composition of pig meat lipids by dietary means. J. Sci. Food Agric..

[5] Dugan MER (2015). Pork as a source of omega-3 (*n*-3) fatty acids. J. Clin. Med..

[6] Kaur G, Cameron-Smith D, Garg M, Sinclair AJ (2011). Docosapentaenoic acid (22:5*n*–3): A review of its biological effects. Prog. Lipid Res..

[7] Ma X, Jiang Z, Lai C (2016). Significance of increasing *n*-3 PUFA content in pork on human health. Crit. Rev. Food Sci. Nutr..

[8] Wood JD, Enser M (1997). Factors influencing fatty acids in meat and the role of antioxidants in improving meat quality. Br. J. Nutr..

[9] Florou-Paneri, P. *et al.* Alternative protein sources to soybean meal in pig diets. *J. Food Agric. Environ*. **12**, 655–660 (2014).

[10] Liu J, Chen F (2016). Biology and industrial applications of *Chlorella*: Advances and prospects. Adv. Biochem. Eng. Biotechnol..

[11] Martins CF (2021). Using microalgae as a sustainable feed resource to enhance quality and nutritional value of pork and poultry meat. Foods.

[12] Safi C, Zebib B, Merah O, Pontalier PY, Vaca-Garcia C (2014). Morphology, composition, production, processing and applications of *Chlorella vulgaris*: A review. Renew. Sustain. Energy Rev..

[13] Kotrbáček V, Doubek J, Doucha J (2015). The chlorococcalean alga *Chlorella* in animal nutrition: a review. J. Appl. Phycol..

[14] Batista AP, Gouveia L, Bandarra NM, Franco JM, Raymundo A (2013). Comparison of microalgal biomass profiles as novel functional ingredient for food products. Algal Res..

[15] Baudelet P-H, Ricochon G, Linder M, Muniglia L (2017). A new insight into cell walls of Chlorophyta. Algal Res..

[16] Teuling E, Wierenga PA, Agboola JO, Gruppen H, Schrama JW (2019). Cell wall disruption increases bioavailability of *Nannochloropsis gaditana* nutrients for juvenile Nile tilapia (*Oreochromis niloticus*). Aquaculture.

[17] Amorim ML (2021). Microalgae proteins: production, separation, isolation, quantification, and application in food and feed. Crit. Rev. Food Sci. Nutr..

[18] Ahmad MT, Shariff M, Md. Yusoff F, Goh YM, Banerjee S (2020). Applications of microalga *Chlorella vulgaris* in aquaculture. Rev. Aquac..

[19] Son J-H, Ravindran V (2012). Feed enzyme technology: Present status and future developments. Recent Patents Food Nutr. Agric..

[20] Zheng H (2011). Disruption of *Chlorella vulgaris* cells for the release of biodiesel-producing lipids: A comparison of grinding, ultrasonication, bead milling, enzymatic lysis, and microwaves. Appl. Biochem. Biotechnol..

[21] Cho HS, Oh YK, Park SC, Lee JW, Park JY (2013). Effects of enzymatic hydrolysis on lipid extraction from *Chlorella vulgaris*. Renew. Energy.

[22] Gerken HG, Donohoe B, Knoshaug EP (2013). Enzymatic cell wall degradation of *Chlorella vulgaris* and other microalgae for biofuels production. Planta.

[23] Coelho D (2019). Novel combination of feed enzymes to improve the degradation of *Chlorella vulgaris* recalcitrant cell wall. Sci. Rep..

[24] Mühlroth A (2013). Pathways of lipid metabolism in marine algae, co-expression network, bottlenecks and candidate genes for enhanced production of EPA and DHA in species of chromista. Mar. Drugs.

[25] Kasper C (2020). Omics application in animal science—a special emphasis on stress response and damaging behaviour in pigs. Genes.

[26] Valdés A, Cifuentes A, León C (2017). Foodomics evaluation of bioactive compounds in foods. TrAC - Trends Anal. Chem..

[27] Benítez R (2019). Breed, diet, and interaction effects on adipose tissue transcriptome in iberian and duroc pigs fed different energy sources. Genes.

[28] Veiseth-Kent, E., de Almeida, A. M., Picard, B. & Hollung, K. Proteomics in skeletal muscle research. In *Proteomics in Domestic Animals: from Farm to Systems Biology* (eds. Almeida, A. M., Eckersal, D. & Miller, I.) 195–217 (Springer International Publishing, Berlin, 2018). 10.1007/978-3-319-69682-9_10

[29] Ribeiro DM (2021). Influence of dietary *Spirulina* inclusion and lysozyme supplementation on the *longissimus lumborum* muscle proteome of newly weaned piglets. J. Proteomics.

[30] Paredi G, Raboni S, Bendixen E, de Almeida AM, Mozzarelli A (2012). ‘Muscle to meat’ molecular events and technological transformations: The proteomics insight. J. Proteomics.

[31] Sirri R (2018). Effect of diets supplemented with linseed alone or combined with vitamin E and selenium or with plant extracts, on *Longissimus thoracis* transcriptome in growing-finishing Italian Large White pigs. J. Anim. Sci. Biotechnol..

[32] Yang H, Xu X, Ma H, Jiang J (2016). Integrative analysis of transcriptomics and proteomics of skeletal muscles of the Chinese indigenous Shaziling pig compared with the Yorkshire breed. BMC Genet..

[33] Liu S, Han W, Jiang S, Zhao C, Wu C (2016). Integrative transcriptomics and proteomics analysis of *longissimus dorsi* muscles of Canadian double-muscled Large White pigs. Gene.

[34] Coelho D (2020). A high dietary incorporation level of *Chlorella vulgaris* improves the nutritional value of pork fat without impairing the performance of finishing pigs. Animals.

[35] Liao Y, Smyth GK, Shi W (2014). FeatureCounts: An efficient general purpose program for assigning sequence reads to genomic features. Bioinformatics.

[36] Canelli G (2021). Tailored enzymatic treatment of *Chlorella vulgaris* cell wall leads to effective disruption while preserving oxidative stability. Lwt.

[37] Tibbetts SM, MacPherson T, McGinn PJ, Fredeen AH (2016). *In vitro* digestion of microalgal biomass from freshwater species isolated in Alberta, Canada for monogastric and ruminant animal feed applications. Algal Res..

[38] Martins CF (2022). Influence of *Chlorella vulgaris* on growth, digestibility and gut morphology and microbiota of weaned piglet. Sci. Rep..

[39] Skugor A (2019). Effects of long-term feeding of rapeseed meal on skeletal muscle transcriptome, production efficiency and meat quality traits in Norwegian Landrace growing-finishing pigs. PLoS One.

[40] Jiang Z (2008). Significant associations of stearoyl-CoA desaturase (*SCD1*) gene with fat deposition and composition in skeletal muscle. Int. J. Biol. Sci..

[41] Paton CM, Ntambi JM (2009). Biochemical and physiological function of stearoyl-CoA desaturase. Am. J. Physiol. - Endocrinol. Metab..

[42] Puig-Oliveras, A. *et al.* Differences in muscle transcriptome among pigs phenotypically extreme for fatty acid composition. *PLoS One* **9**, e103668; 10.1371/journal.pone.0103668 (2014). 10.1371/journal.pone.0099720 PMC4057286 24926690

[43] Xu X, So JS, Park JG, Lee AH (2013). Transcriptional control of hepatic lipid metabolism by SREBP and ChREBP. Semin. Liver Dis..

[44] Corominas J (2013). Analysis of porcine adipose tissue transcriptome reveals differences in *de novo* fatty acid synthesis in pigs with divergent muscle fatty acid composition. BMC Genomics.

[45] Edwards, P. A., Tabor, D., Kast, H. R., Venkateswaran, A. Regulation of gene expression by SREBP and SCAP. *Biochim. Biophys. Acta Mol. Cell Biol. Lipids* **1529**, 103–113 (2000). 10.1016/s1388-1981(00)00140-2 11111080

[46] Oczkowicz M (2020). 3′quant mRNA-Seq of porcine liver reveals alterations in UPR, acute phase response, and cholesterol and bile acid metabolism in response to different dietary fats. Genes.

[47] Jing E (2011). Sirtuin-3 (Sirt3) regulates skeletal muscle metabolism and insulin signaling via altered mitochondrial oxidation and reactive oxygen species production. Proc. Natl. Acad. Sci. U. S. A..

[48] Marcus JM, Andrabi SA (2018). Sirt3 regulation under cellular stress: Making sense of the ups and downs. Front. Neurosci..

[49] Palacios, O. M. *et al.* Diet and exercise signals regulate SIRT3 and activate AMPK and PGC-1alpha in skeletal muscle. *Aging (Albany NY)*. **1**, 771–783. 10.18632/aging.100075 (2009). 10.18632/aging.100075 PMC2815736 20157566

[50] Hirschey MD (2010). SIRT3 regulates mitochondrial fatty-acid oxidation by reversible enzyme deacetylation. Nature.

[51] Gudiksen, A., Pilegaard, H. PGC-1α and fasting-induced PDH regulation in mouse skeletal muscle. *Physiol. Rep*. **5**, e13222; 10.14814/phy2.13222 (2017). 10.14814/phy2.13222 PMC5392513 28400503

[52] Ansari A (2017). Function of the SIRT3 mitochondrial deacetylase in cellular physiology, cancer, and neurodegenerative disease. Aging Cell.

[53] Hunt MC, Alexson SEH (2002). The role Acyl-CoA thioesterases play in mediating intracellular lipid metabolism. Prog. Lipid Res..

[54] Hunt MC, Siponen MI, Alexson SEH (2012). The emerging role of acyl-CoA thioesterases and acyltransferases in regulating peroxisomal lipid metabolism. Biochim. Biophys. Acta Mol. Basis Dis..

[55] Ruan D (2021). Weighted single-step GWAS identified candidate genes associated with growth traits in a Duroc pig population. Genes.

[56] Kirkby B, Roman N, Kobe B, Kellie S, Forwood JK (2010). Functional and structural properties of mammalian acyl-coenzyme A thioesterases. Prog. Lipid Res..

[57] Lv Z, Xing K, Li G, Liu D, Guo Y (2018). Dietary genistein alleviates lipid metabolism disorder and inflammatory response in laying hens with fatty liver syndrome. Front. Physiol..

[58] Westin MAK, Hunt MC, Alexson SEH (2005). The identification of a succinyl-CoA thioesterase suggests a novel pathway for succinate production in peroxisomes. J. Biol. Chem..

[59] Hunt MC, Tillander V, Alexson SEH (2014). Regulation of peroxisomal lipid metabolism: The role of acyl-CoA and coenzyme A metabolizing enzymes. Biochimie.

[60] Lim KS (2017). Identification of differentially expressed genes in *longissimus* muscle of pigs with high and low intramuscular fat content using RNA sequencing. Anim. Genet..

[61] Uniprot. ZNF879. (2017). https://www.uniprot.org/uniprot/A0A287AMY9. (Accessed: 1st May 2022)

[62] Lee DH, Kim JE, Kang YJ (2013). Insulin like growth factor binding Protein-5 regulates excessive vascular smooth muscle cell proliferation in spontaneously hypertensive rats via ERK 1/2 phosphorylation. Korean J. Physiol. Pharmacol..

[63] Fan B, Onteru SK, Rothschild MF (2009). The GGT1 and IGFBP5 genes are associated with fat deposition traits in the pig (Brief Report). Arch. Anim. Breed..

[64] Reyer H, Varley PF, Murani E, Ponsuksili S, Wimmers K (2017). Genetics of body fat mass and related traits in a pig population selected for leanness. Sci. Rep..

[65] Madeira MS (2017). Reduced protein diets increase intramuscular fat of *psoas major*, a red muscle, in lean and fatty pig genotypes. Animal.

[66] Janicki B, Buzała M (2013). The role of creatine in the organism of pigs and its effect on the quality of pork: a review / Rola kreatyny w organizmie świń i jej wpływ na jakość mięsa wieprzowego: artykuł przeglądowy. Ann. Anim. Sci..

[67] Zhang J, Gao Y, Lu Q, Sa R, Zhang H (2015). iTRAQ-based quantitative proteomic analysis of *longissimus* muscle from growing pigs with dietary supplementation of non-starch polysaccharide enzymes. J. Zhejiang Univ. B.

[68] Dang-Nguyen TQ (2011). Development of single blastomeres derived from two-cell embryos produced in vitro in pigs. Theriogenology.

[69] Yang F, Wang Q, Wang M, He K, Pan Y (2012). Associations between gene polymorphisms in two crucial metabolic pathways and growth traits in pigs. Chinese Sci. Bull..

[70] Mouslim C, Aittaleb M, Hume RI, Akaaboune M (2012). A role for the calmodulin kinase ii-related anchoring protein (alpha-kap) in maintaining the stability of nicotinic acetylcholine receptors. J. Neurosci..

[71] Guerrero-Castillo S (2017). The assembly pathway of mitochondrial respiratory chain complex I. Cell Metab..

[72] Omi S, Yamanouchi K, Nakamura K, Matsuwaki T, Nishihara M (2019). Reduced fibrillar collagen accumulation in skeletal muscle of secreted protein acidic and rich in cysteine (SPARC)-null mice. J. Vet. Med. Sci..

[73] Eusebi PG (2017). A genome-wide association analysis for carcass traits in a commercial Duroc pig population. Anim. Genet..

[74] Madeira MS (2014). Combined effects of dietary arginine, leucine and protein levels on fatty acid composition and gene expression in the muscle and subcutaneous adipose tissue of crossbred pigs. Br. J. Nutr..

[75] Macaulay IC (2016). Separation and parallel sequencing of the genomes and transcriptomes of single cells using G&T-seq. Nat. Protoc..

[76] Baym M (2015). Inexpensive multiplexed library preparation for megabase-sized genomes. PLoS One.

[77] Chen S, Zhou Y, Chen Y, Gu J (2018). Fastp: An ultra-fast all-in-one FASTQ preprocessor. Bioinformatics.

[78] Chen C (2012). Solexa sequencing identification of conserved and novel microRNAs in backfat of large white and Chinese Meishan pigs. PLoS One.

[79] Bindea G (2009). ClueGO: A Cytoscape plug-in to decipher functionally grouped gene ontology and pathway annotation networks. Bioinformatics.

[80] Osório H (2021). Proteomics analysis of gastric cancer patients with diabetes mellitus. J. Clin. Med..

[81] Deutsch EW (2017). The ProteomeXchange consortium in 2017: Supporting the cultural change in proteomics public data deposition. Nucleic Acids Res..

[82] Vizcaíno JA (2016). 2016 update of the PRIDE database and its related tools. Nucleic Acids Res..

[83] Singh A (2019). DIABLO: An integrative approach for identifying key molecular drivers from multi-omics assays. Bioinformatics.

