# Supplementary material for: Integrated Omics analysis of pig muscle metabolism under the effects of dietary Chlorella vulgaris and exogenous enzymes
Source: Sci Rep. 2022 Oct 10;12:16992. doi: 10.1038/s41598-022-21466-z (PMC9551059; doi:10.1038/s41598-022-21466-z)

**Integrated Omics analysis of pig muscle metabolism under the effects of dietary *Chlorella vulgaris* and exogenous enzymes**

Diogo Coelho^1,2, +^, David Ribeiro^3, +^, Hugo Osório^4,5,6^, André Martinho de Almeida^3^, José António Mestre Prates^1,2 *^

^1^ CIISA - Centro de Investigação Interdisciplinar em Sanidade Animal, Faculdade de Medicina Veterinária, Universidade de Lisboa, Alto da Ajuda, 1300-477 Lisboa, Portugal.

^2^ Laboratório Associado para Ciência Animal e Veterinária (AL4AnimalS).

^3^ LEAF - Linking Landscape, Environment, Agriculture and Food Research Center, Associated Laboratory TERRA, Instituto Superior de Agronomia, Universidade de Lisboa, Tapada da Ajuda, 1349-017 Lisboa, Portugal

^4^ i3S - Instituto de Investigação e Inovação em Saúde, Universidade do Porto, 4200-135 Porto, Portugal.

^5^ IPATIMUP - Institute of Molecular Pathology and Immunology of the University of Porto, Universidade do Porto, 4200-135 Porto, Portugal

^6^ Departamento de Patologia, Faculdade de Medicina, Universidade do Porto, 4200-319 Porto, Portugal

^*^ Correspondence and requests for material should be addressed to J.A.M.P. (email [japrates@fmv.utl.pt](mailto:japrates@fmv.ulisboa.pt))

^+^ Both authors contributed equally to this paper.

**Supplementary Table S1.** Identification of animal samples and respective experimental diets, complete list of reads obtained from RNA sequencing output and percentage of mapping in the reference genome of *Sus scrofa*.

| **Sample** | **Diet** | **Raw reads** | **Trimmed reads** | **Mapped (%)** | **Unmapped (%)** | **Multiple Mapped (%)** | **Uniquely Mapped (%)** |
| --- | --- | --- | --- | --- | --- | --- | --- |
| 4S | Control | 136580949 | 117259648 | 91.77 | 8.23 | 14.72 | 77.05 |
| 12S | Control | 51879432 | 7795011 | 84.22 | 15.78 | 21.31 | 62.9 |
| 16S | Control | 88432513 | 41741016 | 91.24 | 8.76 | 20 | 71.24 |
| 28S | Control | 63334886 | 60044077 | 86.62 | 13.38 | 12.49 | 74.13 |
| 32S | Control | 23504112 | 22640765 | 90.99 | 9.01 | 9.13 | 81.85 |
| 36S | Control | 27110688 | 21838310 | 92.34 | 7.66 | 11.16 | 81.18 |
| 7S | CV | 38639268 | 37048023 | 91.44 | 8.56 | 8.89 | 82.55 |
| 15S | CV | 60416183 | 57904565 | 91.68 | 8.32 | 4.76 | 86.91 |
| 19S | CV | 43862260 | 42197540 | 89.82 | 10.18 | 8.37 | 81.45 |
| 31S | CV | 25174342 | 24326643 | 90.56 | 9.44 | 7.4 | 83.16 |
| 35S | CV | 31857992 | 30339024 | 85.55 | 14.45 | 8.79 | 76.77 |
| 39S | CV | 33030460 | 14280318 | 91.13 | 8.87 | 14.36 | 76.77 |
| 6S | CV+R | 21793305 | 21313154 | 91.53 | 8.47 | 12.07 | 79.46 |
| 10S | CV+R | 28421935 | 27172124 | 88.8 | 11.2 | 10.93 | 77.87 |
| 18S | CV+R | 39009823 | 38057887 | 92.4 | 7.6 | 6.81 | 85.59 |
| 22S | CV+R | 25068007 | 24507520 | 92.15 | 7.85 | 6.88 | 85.27 |
| 26S | CV+R | 33112067 | 32297526 | 92.65 | 7.35 | 7.16 | 85.49 |
| 34S | CV+R | 42807058 | 41264107 | 91.17 | 8.83 | 6.4 | 84.77 |
| 1S | CV+M | 24857044 | 16680808 | 92.4 | 7.6 | 11.3 | 81.1 |
| 5S | CV+M | 44932627 | 43324278 | 91.52 | 8.48 | 10.53 | 80.99 |
| 9S | CV+M | 39978061 | 38466718 | 90.22 | 9.78 | 9.75 | 80.47 |
| 21S | CV+M | 20114052 | 19418134 | 88.32 | 11.68 | 9.31 | 79.01 |
| 29S | CV+M | 29567990 | 28469992 | 89.93 | 10.07 | 8.48 | 81.45 |
| 33S | CV+M | 24399759 | 16200536 | 91.53 | 8.47 | 16.62 | 74.91 |

**Supplementary Figure S1.** Relative abundance (%) of the mapped reads along the different regions of the *Sus scrofa* reference genome.


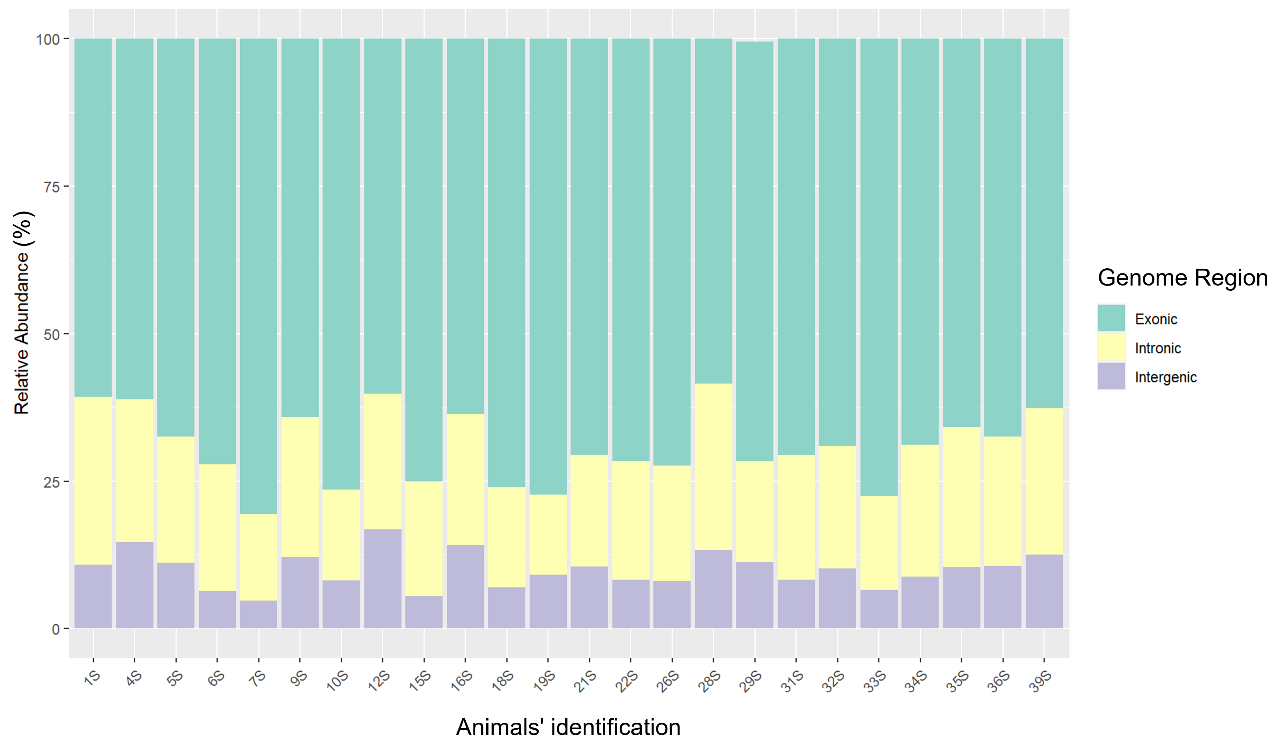


**Supplementary Figure S2.** Top ten most abundantly expressed protein-coding genes of pig muscle transcriptome from all sampled animals of each experimental diets. Control (a cereal and soybean meal-based diet); CV (control diet with 5% *C. vulgaris*); CV+R (CV diet supplemented with 0.005% of Rovabio^®^ Excel AP) and CV+M (CV diet supplemented with 0.01% of the preselected four-CAZyme mixture).


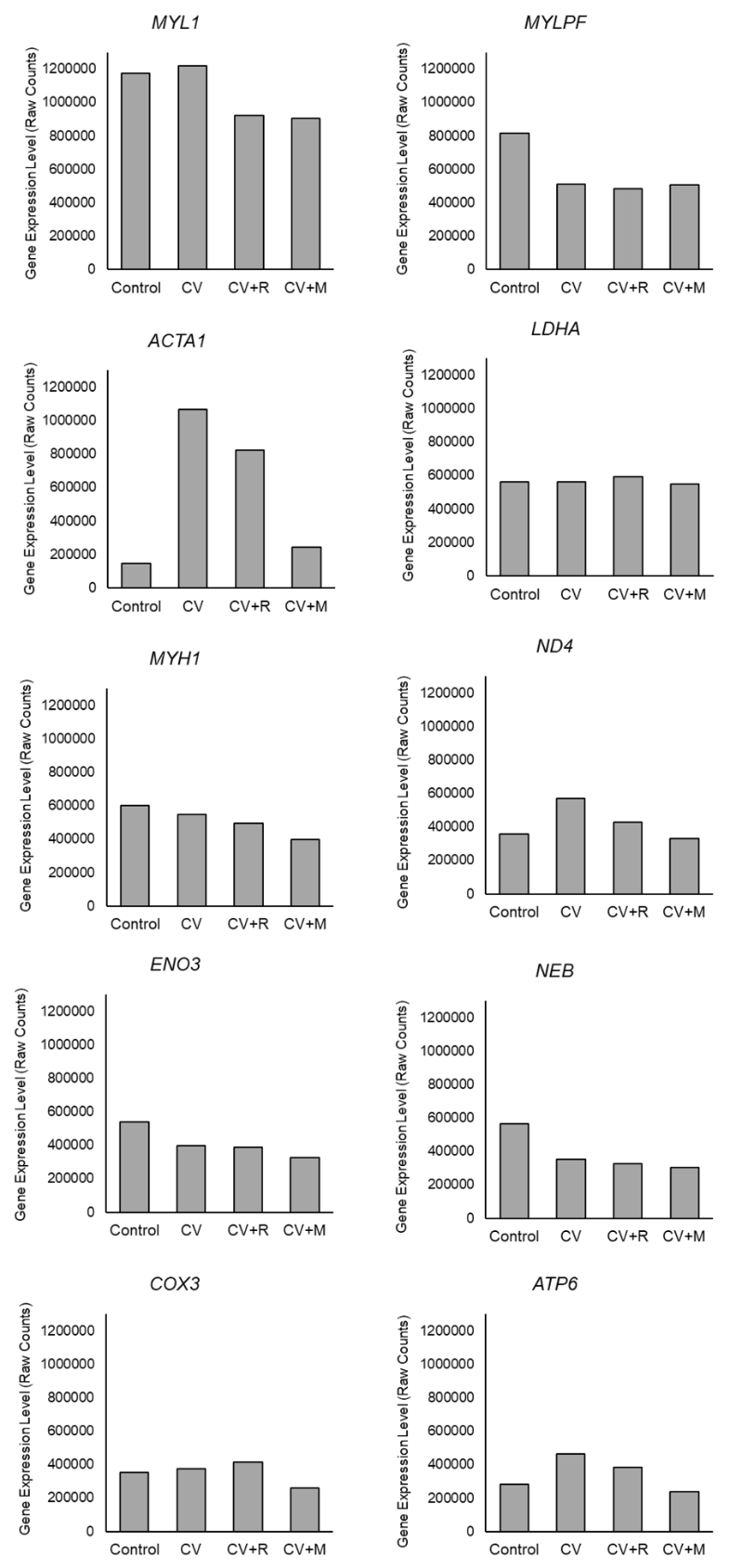


**Supplementary Table S2.** Differential protein abundance in Control vs CV (5% dietary *C. vulgaris*).

| **UniProt Accession** | **Description** | **Gene** | **Unique peptides** | **Fold Change** | **p-value** |
| --- | --- | --- | --- | --- | --- |
| Higher in CV *vs* control | | | | | |
| A0A5G2QVS9 | Uncharacterized protein | MYL1 | 2 | 8.93 | <0.0001 |
| A0A286ZJN9 | Acyl-CoA binding domain containing 7 | ACBD7 | 3 | 6.89 | <0.0001 |
| A0A287BKR9 | Uncharacterized protein | LOC100157017 | 2 | 5.55 | 0.0015 |
| F1SL24 | Tetraspanin | CD9 | 2 | 4.53 | 0.0002 |
| F1S288 | Syntrophin beta 1 | SNTB1 | 2 | 3.95 | 0.0193 |
| F2Z5G8 | 40S ribosomal protein S25 | RPS25 | 2 | 3.74 | 0.0017 |
| A0A5G2QP96 | Uncharacterized protein | SSR1 | 2 | 3.62 | 0.0024 |
| A0A287A5S7 | Dimethylarginine dimethylaminohydrolase 2 | DDAH2 | 2 | 3.41 | 0.0441 |
| P20112 | SPARC | SPARC | 2 | 3.35 | 0.0453 |
| Q75NG7 | Troponin T fast skeletal muscle type | tnnt3 | 2 | 3.05 | 0.0133 |
| A0A5G2Q7Z2 | Protein S100 | LOC110258323 | 2 | 2.71 | 0.0368 |
| Higher in Control *vs* CV | | | | | |
| F1SS64 | Myosin-2 | MYH2 | 15 | 0.31 | 0.0352 |
| A0A286ZNH3 | 2-iminobutanoate/2-iminopropanoate deaminase | RIDA | 4 | 0.31 | 0.0333 |
| A0A287BFN4 | HBS1 like translational GTPase | HBS1L | 3 | 0.28 | 0.0136 |
| I3LLY3 | Uncharacterized protein | ACTN1 | 2 | 0.26 | 0.0061 |
| A0A287AR93 | Protein kinase C and casein kinase substrate in neurons 3 | PACSIN3 | 8 | 0.21 | 0.0006 |
| Q29092 | Endoplasmin | HSP90B1 | 4 | 0.18 | 0.0258 |
| I3LIE7 | Myosin-binding protein H | MYBPH | 12 | 0.18 | 0.0001 |
| P62802 | Histone H4 | NA | 3 | 0.18 | 0.0008 |
| F1RVC9 | Heterogeneous nuclear ribonucleoprotein D | HNRNPD | 2 | 0.01 | <0.0001 |
| K7GP28 | Hydroxysteroid 17-beta dehydrogenase 10 | HSD17B10 | 2 | 0.01 | <0.0001 |

**Supplementary Table S3.** Differential protein abundance in Control vs CV+R (5% dietary *C. vulgaris* + 0.005% Rovabio^®^ Excel AP).

| **UniProt Accession** | **Description** | **Gene** | **Unique peptides** | **Fold Change** | **p-value** |
| --- | --- | --- | --- | --- | --- |
| Higher in CV+R *vs* Control | | | | | |
| A0A287BKR9 | Uncharacterized protein | LOC100157017 | 2 | 7.67 | <0.0001 |
| F1S288 | Syntrophin beta 1 | SNTB1 | 2 | 4.72 | 0.0240 |
| K7GP28 | Hydroxysteroid 17-beta dehydrogenase 10 | HSD17B10 | 2 | 4.26 | 0.0197 |
| F1RNZ1 | Ubiquinol-cytochrome c reductase, Rieske iron-sulphur polypeptide 1 | UQCRFS1 | 2 | 4.23 | 0.0065 |
| F1RJH2 | Short-chain-specific acyl-CoA dehydrogenase, mitochondrial | ACADS | 2 | 3.54 | 0.0276 |
| Higher in Control vs CV+R | | | | | |
| Q9TV62 | Myosin-4 | MYH4 | 2 | 0.40 | 0.0404 |
| A0A287BPU2 | Nebulin | NEB | 2 | 0.37 | 0.0166 |
| I3LMU6 | Reticulocalbin 3 | RCN3 | 3 | 0.37 | 0.0221 |
| P79293 | Myosin-7 | MYH7 | 75 | 0.36 | 0.0192 |
| Q29577 | Creatine kinase U-type, mitochondrial | CKMT1 | 2 | 0.36 | 0.0292 |
| F1SGP8 | Reticulocalbin-1 | RCN1 | 4 | 0.34 | 0.0060 |
| A0A5G2R1K3 | Sodium/potassium-transporting ATPase subunit alpha | ATP1A3 | 2 | 0.33 | 0.0036 |
| Q9TV61 | Myosin-1 | MYH1 | 9 | 0.32 | 0.0069 |
| A0A287ATJ4 | Alpha-crystallin B chain | CRYAB | 5 | 0.32 | 0.0026 |
| A0A287AXY6 | Synaptosome associated protein 29 | SNAP29 | 2 | 0.26 | 0.0006 |
| F1RVC9 | Heterogeneous nuclear ribonucleoprotein D | HNRNPD | 2 | 0.26 | 0.0008 |
| F1SS64 | Myosin-2 | MYH2 | 15 | 0.25 | 0.0006 |
| I3LNG5 | Calcium/calmodulin dependent protein kinase II alpha | CAMK2A | 2 | 0.09 | <0.0001 |

**Supplementary Table S4.** Differential protein abundance in Control vs CV+M (5% dietary *C. vulgaris* + 0.01% CAZyme mix).

| **UniProt Accession** | **Description** | **Gene** | **Unique peptides** | **Fold Change** | **p-value** |
| --- | --- | --- | --- | --- | --- |
| Higher in CV+M *vs* Control | | | | | |
| A0A5G2QVS9 | Uncharacterized protein | MYL1 | 2 | 23.322 | <0.0001 |
| F1SCC6 | SERPIN domain-containing protein | SERPINA3-2 | 3 | 7.219 | 0.0173 |
| Q6QAP7 | 40S ribosomal protein S17 | RPS17 | 3 | 6.808 | 0.0233 |
| A0A287B182 | Transmembrane protein 263 | TMEM263 | 2 | 6.517 | 0.0284 |
| P20112 | SPARC | SPARC | 2 | 6.145 | 0.0370 |
| Higher in Control *vs* CV+M | | | | | |
| F2Z5S8 | Tubulin alpha chain | TUBA4A | 2 | 0.14 | 0.0240 |
| I3LNG5 | Calcium/calmodulin dependent protein kinase II alpha | CAMK2A | 2 | 0.106 | 0.0058 |
| I3LIE7 | Myosin-binding protein H | MYBPH | 12 | 0.098 | 0.0037 |
| P62802 | Histone H4 |  | 3 | 0.061 | 0.0002 |
| Q29577 | Creatine kinase U-type, mitochondrial | CKMT1 | 2 | 0.052 | 0.0001 |
| I3LLY3 | Uncharacterized protein | ACTN1 | 2 | 0.018 | <0.0001 |
| A0A286ZNN4 | NADH dehydrogenase ubiquinone iron-sulphur protein 3, mitochondrial | NDUFS3 | 3 | 0.01 | <0.0001 |

**Supplementary** **Figure S3.** Circosplot obtained for A: Control vs CV, B: Control vs CV+R and C: Control vs CV+M comparisons using the first two principal components. Yellow: gene names in lower case. Red: protein accession numbers. Figure was obtained using the circosPlot function of the mixOmics package in R, with a threshold of 0.7.


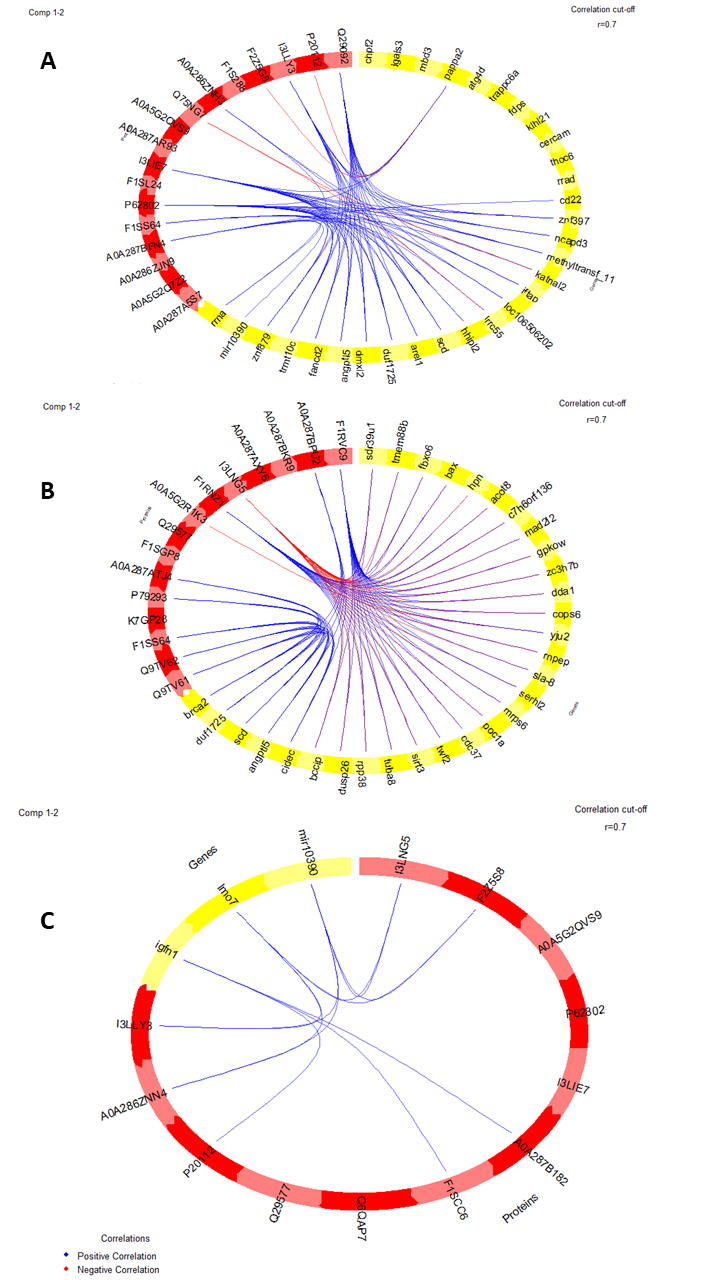


**Supplementary** **Figure S4.** Correlation (Pearson coefficient) between the first principal component of each dataset (differentially regulated transcriptome and proteome) in each comparison: Control vs CV (A), Control vs CV+R (B) and Control vs CV+M (C). Figures were obtained using the plotDiablo function of the mixOmics package in R.


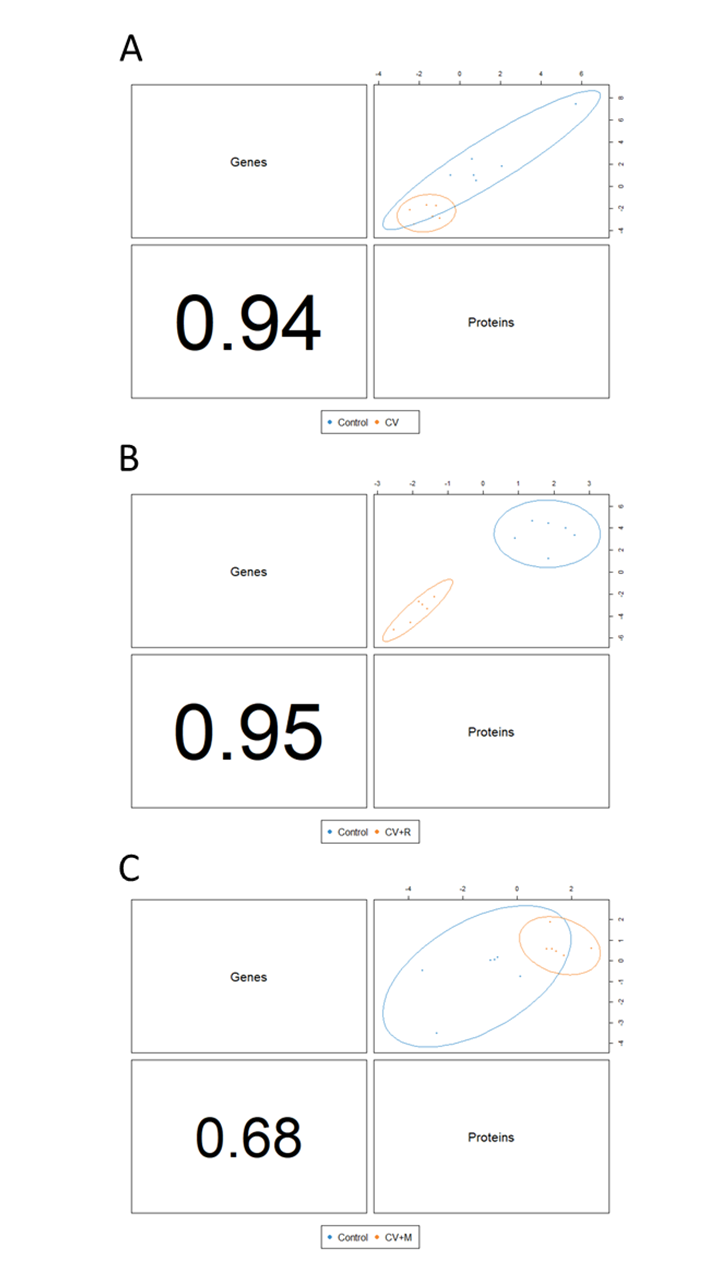


**Supplementary** **Figure S5.** Correlation circle plots obtained using the first two principal components of the N-integration with sparse Discriminant Analysis (Diablo) carried out for each comparison: Control vs CV (A), Control vs CV+R (B) and Control vs CV+M (C). Figures were obtained using the block.splsda function of the mixOmics package in R. Orange: protein accession numbers. Blue: gene names in lower case.


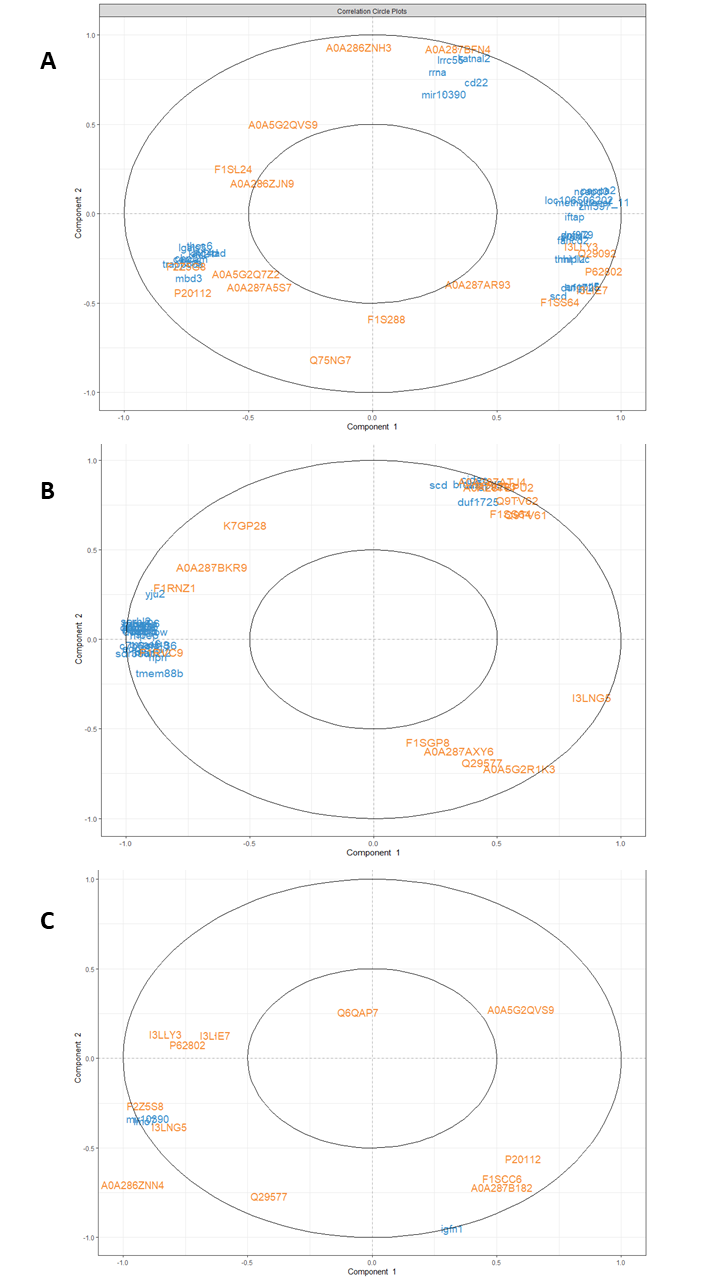

Supplement: Supplementary file 1 — Supplementary Information 1. [file 41598_2022_21466_MOESM1_ESM.docx]
